# Foods of concern, cardiopreventive medication use and risk of cardiovascular diseases: a prospective study in the CARTaGENE cohort

**DOI:** 10.1016/j.ajcnut.2026.101234

**Published:** 2026-02-06

**Authors:** Lise Leblay, Jacob Lessard-Lord, Neha Khandpur, Jean-Sébastien Paquette, Jean-Philippe Drouin-Chartier

**Affiliations:** 1 Centre Nutrition, Santé et Société (NUTRISS), Institut sur la Nutrition et les Aliments Fonctionnels (INAF), Université Laval, Québec, QC, Canada; 2 Faculté de Pharmacie, Université Laval, Québec, QC, Canada; 3 Division of Human Nutrition and Health, Wageningen University, The Netherlands; 4 Département de Médecine Familiale et de Médecine D’urgence, Faculté de Médecine, Université Laval, Québec, QC, Canada; 5 VITAM Sustainable Health Research Center, Université Laval, Québec, QC, Canada; 6 Groupe de Médecine de Famille Universitaire du Nord de Lanaudière, Centre intégré de santé et de services sociaux de Lanaudière, Saint-Charles-Borromée, Québec, QC, Canada

**Keywords:** ultraprocessed foods, medication use, cardiovascular disease, front-of-package symbol, hypercholesterolemia, hypertension

## Abstract

**Background:**

In Canada, by 2026, prepackaged foods high in saturated fat, sodium, or sugar require a front-of-package warning symbol (FOPS). However, evidence on ultraprocessed foods (UPFs) raises concerns about whether this approach fully captures all foods of concern—particularly for individuals with hypertension or hypercholesterolemia, for whom dietary changes are crucial. It is also important to assess whether medication use diminishes the benefits of limiting such foods.

**Objectives:**

We examined the joint association of consuming foods of concern—defined as UPFs or foods with FOPS—medication use, and cardiovascular disease (CVD) risk in adults with hypertension and/or hypercholesterolemia from the CARTaGENE cohort.

**Methods:**

This prospective cohort study included 2123 participants free of CVD but with hypertension and/or hypercholesterolemia at baseline (2009–2010). Using food frequency questionnaire (FFQ) data (2012), UPFs were identified using Nova, and foods with FOPS using Health Canada criteria. Cholesterol- and blood pressure-lowering medication use was self-reported. Incident CVDs were identified using administrative databases, from FFQ completion to 31 December, 2021. Associations between intake of foods of concern, medication use, and CVD risk were assessed using multivariable-adjusted Cox proportional hazards models.

**Results:**

In multivariable-adjusted analyses—including medication use, energy intake, and BMI—the hazard ratio (HR) for CVD risk associated with a 10% lower difference in UPF consumption—accompanied by a proportional higher difference in non-UPF—was 0.87 [95% confidence interval (CI): 0.78, 0.97]. For foods with FOPS, the corresponding HR was 0.80 (95% CI: 0.70, 0.93), with no evidence that this estimate differed significantly from that for UPF (*P* = 0.42). There was no evidence of departure from additivity between medication use and the consumption of foods of concern relative to CVD risk.

**Conclusions:**

In individuals with hypertension or hypercholesterolemia, lower consumption of UPFs or foods with FOPS was similarly associated with lower CVD risk, independent of concomitant medication use.

## Introduction

In Canada, 14 adults die from cardiovascular disease (CVD) every hour, with annual costs exceeding $21 billion [1,2]. As a means of reducing this burden through population dietary changes, a new regulation took effect in 2026 in Canada requiring prepackaged foods with excessive levels of saturated fat, sodium, or total sugars (i.e., naturally occurring and/or added) to display a front-of-package nutrition symbol (FOPS) indicating their high content of these nutrients, whose intake has been consistently associated with a higher risk of CVD [3]. Still, beyond the composition of foods in these 3 nutrients, the accumulating data on ultraprocessed food (UPF) consumption and its link to CVD risk [4] raise questions about whether limiting the range of foods of concern to those high in saturated fat, sodium, or sugars, independent of their level of processing, is sufficient. UPF are ready-to-heat/ready-to-eat formulations of processed food substances and cosmetic additives that result from a series of industrial processes [5]. These foods are generally energy-dense, nutrient-poor, and are characterized by their accessibility, availability, and affordability [6,7]. A majority of UPF exhibit excessive content in saturated fat, sodium, or sugars, but also low content in dietary fibers, vitamins, and minerals, and their consumption is associated with lower diet quality and excessive energy intake [[8], [9], [10]].

However, the adverse health effects of UPF may extend beyond their generally poor nutritional composition. Accumulating evidence links processing-related features—such as certain food additive groups—to cardiometabolic diseases, including CVD [11], although recent clinical trial data suggest equivocal short-term effects on traditional cardiometabolic risk factors [12].

The issue of adequately identifying foods of concern is even more critical for individuals with risk-increasing conditions, such as hypertension or high blood cholesterol, given the crucial role of dietary modification in CVD prevention. Furthermore, it is essential to assess whether preventive medication use weakens the benefits of limiting the consumption of unhealthy foods, especially as growing evidence indicates that diet is often overlooked in favor of medication [[13], [14], [15], [16], [17]]. Indeed, our group has generated key data from Canada showing that, in primary CVD prevention, the use of statins, antihypertensive medications, and glucose-lowering drugs impairs diet quality and that adherence to these medications negatively correlates with adherence to healthy dietary recommendations [[13], [14], [15], [16], [17]]. This is concerning because it suggests that drug-based approaches are often preferred over dietary modifications in the primary prevention of CVDs. Assessing these issues will not only provide contemporary evidence on foods of concern for CVDs but also reinforce the need to integrate meaningful dietary changes alongside medication for optimal prevention.

In this study, we examined the joint association between the consumption of foods of concern—defined either as UPFs or as foods requiring a FOPS under the Canadian regulation—medication use—i.e., blood pressure (BP)- and cholesterol-lowering drugs—and the risk of CVDs among adults with hypertension and/or high blood cholesterol. We hypothesized that lower consumption of foods with FOPS or that are UPF is associated with a lower risk of CVDs, without a meaningful difference in the associations between these 2 food categories, and that these associations are not modified by concomitant medication use.

## Methods

The study protocol was reviewed and approved by the Laval University Ethics Committee, the CARTaGENE Sample and Data Access Committee, and the Québec Statistics Institute. All participants provided written informed consent.

### Study population

This study is a prospective cohort study conducted within the CARTaGENE Québec population-based cohort (phase A) [18]. In 2009–2010, residents from the province of Québec (Canada), aged 40–69 y, and living in metropolitan areas representing 56% of the Québec population, were randomly selected from provincial health insurance registries to participate in CARTaGENE. Recruitment was stratified by age, sex, and area of residence according to 2006 census data. A total of 78,036 individuals were contacted. Of these, 20,007 attended the in-person interview and signed the consent form, but not all completed the health questionnaire. The primary CARTaGENE cohort consists of 19,069 participants who completed both the baseline in-person visit and the health questionnaire [18].

Baseline data collection took place during an in-person visit (2009–2010), which included a self-administered sociodemographic and lifestyle questionnaire [19], an interviewer-administered health questionnaire, physical measurements, and biospecimen collection. In 2011–2012, participants were invited to complete a food frequency questionnaire (FFQ) from home. Approximately 10,000 individuals returned the completed FFQ. The FFQ was the Canadian Dietary History Questionnaire II (CDHQII), which assessed diet over the 12 mo preceding its completion (fully described below) [20,21]. Follow-up of CARTaGENE participants for incident disease diagnoses is secured through linkage with health and administrative databases, i.e., the Québec Maintenance and Exploitation of Data for the Study of Hospital Clientele and the Québec Institute of Statistics databases [22,23]. These databases include data for nearly all individuals enrolled in Québec’s universal healthcare program, covering ∼99% of the province’s population (i.e., minimal loss to follow-up) [23]. Both databases were individually linked to the CARTaGENE dataset at the participant level.

For this study, we included participants who returned the FFQ and met the following criteria: *1*) adequately completed the FFQ (i.e., <40% of blank items); *2*) reported plausible energy intakes in the FFQ (i.e., females: 500–3500 kcal/d; males: 800–4200 kcal/d) [24]; *3*) had no history of diabetes, CVD or cancer before FFQ completion; *4*) self-reported a diagnosis and/or treatment for high BP and/or high blood cholesterol (i.e., all included participants were aware that they had high BP and/or high blood cholesterol). A previous study reported excellent agreement between self-report of medical diagnosis in CARTaGENE health questionnaire and administrative health databases [23]. A total of 2123 individuals were included in the study (Supplementary Figure 1).

### Assessment of diet and intakes of foods of concern

Dietary intakes were assessed using the CDHQII [20,21,25,26], an FFQ that evaluates the frequency and portion sizes of foods consumed during the 12 mo preceding its completion. The CDHQII was originally developed and validated by the US National Cancer Institute [21,[27], [28], [29]], and has since been adapted for the Canadian context to reflect local food availability, brand names, nutrition composition and food fortification. For food composition (e.g., sodium, sugars, and saturated fat), the CDHQII was linked to the Canadian Nutrient File database [21].

**FIGURE 1:**
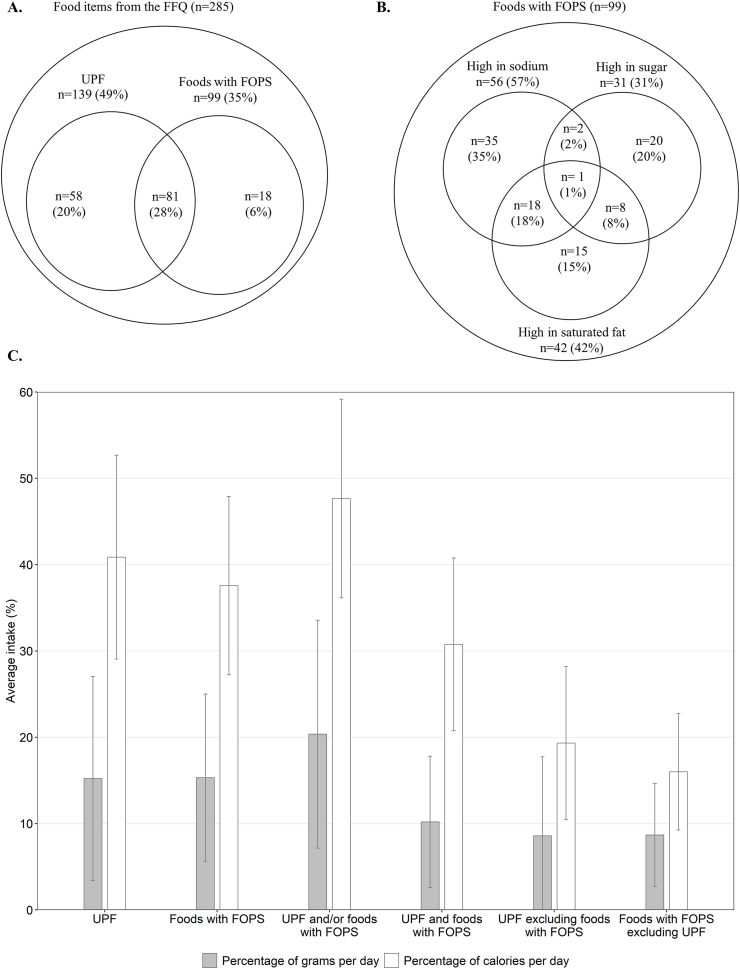
(A) Number of food items classified as UPF and/or displaying a FOPS among the 285 items in the Canadian Dietary History Questionnaire II. (B) Number of food items meeting the criteria for saturated fat, sodium, and/or sugar among the 99 items with a FOPS. (C) Average intake of foods of concern among the 2023 participants included in the study. FFQ, food frequency questionnaire; FOPS, front-of-package nutrition symbol; UPF, ultraprocessed foods.

Each food and beverage item from the FFQ was first categorized as ultraprocessed or not according to the Nova classification [6,30,31]. Nova classifies foods on the basis of the extent and purpose of the industrial processing they undergo and accounts for the physical, biological, and chemical methods used in their manufacture, including the use of additives. The recently published best practices for applying the Nova food classification system were used [32]. The multistep approach previously developed by 2 authors (NK, J-PD-C) was also used [30]. Briefly, a food list compiling all 285 single-ingredient and multi-ingredient food items from the CDHQII was created. The investigators had access to ingredient lists of multi-ingredient food items. In these cases, the item was analyzed at the ingredient level. When ≥1 ingredient was considered as UPF, the food item was consequently also considered UPF. For manufactured food items, the list of ingredients from the top 5 corresponding products on a major national grocery retailer website was used as a reference [32]. If ≥3 of the top 5 products listed on the website were UPF, the food item was categorized as UPF [32]. Finally, the processing level of some food items remained uncertain, mostly because they could be homemade culinary preparations or industrially manufactured products. In primary analyses, these foods were considered as UPF. In sensitivity analyses, these food items were reclassified as non-UPF. The full classification is presented in Supplementary Table 1. A total of 139 items were considered as UPF (Figure 1A). In sensitivity analyses, food items with an uncertain level of processing were reclassified as non-UPF, decreasing the total number to 118 UPF items (Supplementary Figure 2).

Each food and beverage item from the FFQ was also categorized on whether they would require a FOPS according to Health Canada regulation [3]. As of 1 January, 2026, Canada’s FOPS is required on prepackaged foods that meet or exceed specified thresholds for sodium, sugars, or saturated fat. For prepackaged foods that are not main dishes and have a reference amount >30 g or 30 mL, the symbol is triggered at ≥15% of the daily value (DV); for products with a reference amount ≤30 g or 30 mL, it is triggered at ≥10% DV. For prepackaged main dishes with a reference amount ≥200 g, it is triggered at ≥30% DV.

The food item list was cross-referenced with the CDHQII nutritional database comprising information on saturated fat, sodium, and sugar content for prespecified quantities, in grams. The first step was to identify food items that were exempted for the FOPS. For instance, raw single-ingredient meat, homemade items, poultry meat or poultry meat by-products that are not ground or oil and fats are exempted [3]. Next, saturated fat, sodium, and sugar content were compared with the reference thresholds defined by Health Canada. The regulation stipulates that if the content in ≥1 of the 3 nutrients of concern exceeds the threshold, the FOPS is required. As such, a food item was classified as a food with a FOPS if its content in saturated fat, sodium, or sugars exceeded the specified thresholds [3]. As for UPF, the requirement of FOPS for some food items remained uncertain, mostly because they could be homemade or prepackaged foods. In primary analyses, these foods were considered as FOPS. In sensitivity analyses, these food items were reclassified as non-FOPS. The classification is presented in Supplementary Table 1.

A total of 99 items were classified as foods with FOPS (Figure 1A), and 81 of these were classified both as UPF and foods with FOPS. Sodium was the most exceeded criterion for FOPS eligibility, followed by saturated fat and sugar (Figure 1B). In sensitivity analyses, food items with uncertain symbol eligibility were reclassified as non-FOPS, decreasing the total number to 79 foods with FOPS, and 64 overlapping UPF and FOPS (Supplementary Figure 2).

The UPF and FOPS classifications were undertaken using an iterative fashion. A single author (LL) performed the initial classification for UPF and FOPS, which was subsequently verified by 2 authors (NK and J-PD-C), and any conflicting or uncertain classifications were resolved through discussions until agreement was reached.

In primary analyses, consumption of foods of concern was quantified as grams relative to total grams of diet per day (percentage of grams/day). This is intended to consider contribution of low- or noncalorie food items to the overall dietary intakes. In sensitivity analyses, consumption was quantified as a percentage of calories per day [33].

### Assessment of cardiopreventive medication use

At the baseline interview, participants were asked to bring all active prescribed medication [18,34]. Information on BP- and cholesterol-lowering medication use was thus obtained from the health questionnaire. A previous study within CARTaGENE demonstrated the high agreement between self-reported medication and claim prescription records (κ > 0.80) [34]. The use of BP- and/or cholesterol-lowering medication was identified by cross-referencing participant medication list with the anatomical therapeutic chemical classification (i.e., C02, C03, C07, C08, C09, C10AA, C10AX09).

### Assessment of incident CVDs

The primary outcome was incident CVDs, defined as a composite of a first stroke, myocardial infarction, or CVD death. This information was obtained from Québec administrative health databases compiling diagnostic, procedure codes, and death causes per the International Classification of Diseases (ninth and 10th revisions) [35]. Events were identified using algorithms specifically validated for use and surveillance in the Québec administrative health databases by the Quebec National Institute of Public Health [[36], [37], [38]]. Codes for myocardial infarction included 410–414, I20.x–I25.x. Codes for stroke included 3623, 430, 431, 434.x, 436, and 435.x, I60.x, I61.x, I63.x (excluding I63.6), I64, H340, H341, G45.x (excluding G45.4) [[36], [37], [38], [39]]. In the Québec Institute of Statistics database, deaths from myocardial infarction or stroke were identified using these codes, considering both primary and secondary causes of death.

### Assessment of covariables

Participants’ sex (female and male) was reported in the CARTaGENE health questionnaire during the baseline in-person interview. Data on age, household annual income, smoking status, and level of physical activity were collected through the self-administered questionnaire during the in-person interview. Physical activity was assessed using the International Physical Activity Questionnaire [40]. Anthropometry measurements were taken by a research associate during the interviews, including waist circumference (measured twice with a SECA 200 measuring tape), height (measured twice with a SECA 214 portable stadiometer), and weight (measured with a digital scale). Data on alcohol consumption (g/d) and energy intake (kcal/d) were calculated from CDHQII data.

### Power considerations

With 2123 participants and 179 CVD events, under typical variability in consumption of foods of concern, 80% power at 2-sided *α* = 0.05 corresponds to a minimum detectable hazard ratio (HR) of ∼0.81 per 10% lower difference in intake.

### Statistical analyses

Statistical analyses were performed using SAS Studio software (version 3.5). All statistical tests were 2-sided with a significance threshold set at *P* < 0.05. We calculated each participant’s person-years from the date of FFQ completion to the date of a cardiovascular event, death (as a censoring event), or the end of the follow-up (31 December, 2021; also a censoring event), whichever occurred first. Given the low incidence of CVD events during follow-up, HRs were interpreted as approximations of relative risk.

First, Cox proportional hazards regression models were used to evaluate the association between consumption of foods of concern and CVD risk. Analyses were conducted separately for *1*) UPF, *2*) foods with FOPS, *3*) items that were classified as UPF and/or foods with FOPS, and *4*) items that classified as both UPF and with FOPS, expressed in percentage of grams per day. The continuous association was tested to calculate the risk of cardiovascular events for each 10% lower difference in consumption of these foods, treated as a continuous variable. The linearity assumption for continuous covariates was assessed using martingale residual plots. To further evaluate the appropriateness of the linear specification, intake of foods of concern was also categorized into tertiles. Participants in the third tertile of consumption were used as the reference group to inform on differences in CVD risk associated with lower consumption, and HRs and 95% confidence intervals (CIs) were calculated for CVD risk across each tertile of UPF consumption. Survival curves were generated using the "baseline" statement in the SAS "PHREG" procedure.

Model 1 was adjusted for nonmodifiable risk factors [i.e., age and sex (female and male)], household income (<$50,000; $50,000–<$100,000; ≥$100,000) as a surrogate of socioeconomic status, and lifestyle/modifiable risk factors [i.e., smoking status (never, past, and current), physical activity level (low, moderate, and high), alcohol consumption (g/d), hypertension status (none, unmedicated, and medicated), and high blood cholesterol status (none, unmedicated, and medicated)]. Energy intake (kcal/d) and BMI (kg/m^2^) were both considered to lie on the causal pathway between intake of foods of concern and CVD risk. They were therefore added sequentially in model 2 (model 1 + energy intake) and model 3 (model 2 + BMI). The number of missing values for each covariable is presented in Supplementary Table 2. Given the very low frequency of missingness, missing data were imputed using the median for continuous variables and the mode for categorical variables.

In the results figures, we present data from model 3. Results from all models are provided in the supplementary material. To assess whether the associations between the consumption of UPF, foods with FOPS, food items classified as UPF and/or foods with FOPS, and food items classified as both UPF and foods with FOPS differed in relation to CVD risk, HRs were compared using t-tests [41]. Finally, we calculated e-values to determine the minimum strength of association on the risk ratio scale that an unmeasured confounder would need to have with both the diet and CVD to fully explain away our results.

In sensitivity analyses, primary analyses were first repeated by reclassifying food items with an uncertain level of processing as non-UPF and food items with uncertain FOPS eligibility as non-FOPS, and by modeling consumption of foods of concern in percentage of calories per day. Also, the association of foods of concern consumption with CVD risk was tested by sequentially restricting the sample to participants with *1*) hypertension and *2*) high blood cholesterol.

Second, Cox proportional hazards regression models were used to evaluate the associations between medication status (unmedicated compared with medicated) and CVD risk separately among individuals with hypertension and those with high blood cholesterol, as a necessary step before assessing interactions between medication use and diet in relation to CVD risk [42]. The participants with the unmedicated condition were used as the reference group. These models were adjusted for age, sex, smoking status, energy intake, UPF intake, alcohol consumption, physical activity, BMI and concomitant high blood cholesterol (none, unmedicated, medicated), for analyses on medicated hypertension, or concomitant high BP (none, unmedicated, medicated), for analyses on medicated high blood cholesterol. To inform on the adequacy of treatments, we complemented the prospective analyses with cross-sectional analyses on the associations between medication status and BP (mmHg) or LDL cholesterol (mmol/L), using linear regression models (generalized linear model procedure). Systolic BP, diastolic BP, and LDL cholesterol were included as dependent variables in separate models.

Third, Cox proportional hazards regression models were used to examine the joint associations between the consumption of foods of concern (per 10% lower difference in intake), medication status (unmedicated, medicated), and CVD risk. These analyses were conducted separately among individuals with hypertension and among individuals with high blood cholesterol. The models were adjusted for age, sex, household income, smoking status, physical activity level, alcohol consumption, energy intake, BMI, concomitant high blood cholesterol status, for analyses among individuals with hypertension, or concomitant hypertension status, for analyses on individuals with hypercholesterolemia. An interaction term between the foods of concern variable (per 10% lower difference in intake) and medication status (unmedicated, medicated) was included. The *P* value derived from the interaction term reflects interaction between diet and medication use on the multiplicative scale [42]. To evaluate interaction on the additive scale, we calculated the relative excess risk due to interaction (RERI) and the attributable proportion due to interaction (API) [42]. In the present context—where both exposures are hypothesized to be associated with a lower risk of CVD (i.e., HRs for foods of concern are calculated for a 10% lower difference in intake)—the interpretation of these metrics is as follows: *1*) RERI<0 and/or API<0 suggest that the joint association is more protective than expected under additivity (consistent with a synergistic association); *2*) RERI=0 and/or API=0 indicate no departure from additivity; *3*) RERI>0 and/or API>0 suggest that the joint association is less protective than expected under additivity (consistent with an antagonistic association) [42].

## Results

### Characteristics of the study sample

Among the 2123 participants included in the study, 1258 reported having hypertension (283 were unmedicated and 975 were medicated) and 1334 reported having high blood cholesterol (621 were unmedicated and 713 were medicated) (Supplementary Table 3). Characteristics of the 2123 participants included in the study relative to the 730 that met the inclusion/exclusion criteria but did not complete the FFQ are presented in Supplementary Table 4. With the exception of sex distribution, the included sample being ∼50% female and 50% male, compared with 42% female and 58% male in the nonincluded group, differences between groups are minimal. In the sample of 2123 individuals included in the study, total UPF consumption accounted for an average of 15.2% (SD: ±11.8%) of daily diet weight (in grams) and 40.9% (±11.8%) of daily energy intake (Figure 1C). Foods with FOPS contributed, on average, to 15.3% (±9.7%) of total diet weight and 37.6% (±10.3%) of daily caloric intake. The characteristics of the 2123 participants according to tertiles of UPF and foods with FOPS consumption are presented in Table 1. The characteristics of participants were very similar when divided based on UPF or foods with FOPS intake. Participants in tertile 3 were more likely to be males than females, whereas the opposite was observed in tertile 1. The prevalence of hypertension was higher among participants in tertile 1 compared with those in tertile 3, but the prevalence of high blood cholesterol was lower in tertile 1. The number of participants using both antihypertensive and cholesterol-lowering medications was the lowest in tertile 1. Energy intake was higher among participants in tertile 3 compared with those in tertiles 2 and 1. Saturated fat and sodium intakes, but not that of sugar, were positively associated with consumption of foods of concern.

**TABLE 1:** Characteristics of participants according to tertiles of foods of concern consumption^1^

Characteristics	Ultraprocessed foods			Foods with front-of-package nutrition symbol		
Tertile (range of intake in percentage of grams per day)	Tertile 3 (92.4–16.3)	Tertile 2 (<16.3–9.1)	Tertile 1 (<9.1–0.5)	Tertile 3 (92.9–17.4)	Tertile 2 (<17.4–10.3)	Tertile 1 (<10.3–0.6)
Participants, *n* (%)	722 (34.0)	700 (33.0)	701 (33.0)	722 (34.0)	700 (33.0)	701 (33.0)
Ultraprocessed food intake, percentage of grams per day	27.4 ± 12.5	12.4 ± 2.0	5.5 ± 2.3	22.1 ± 11.6	14.5 ± 9.5	8.9 ± 10.3
Foods with front-of-package nutrition symbol, percentage of grams per day	22.3 ± 10.7	15.4 ± 5.5	8.0 ± 5.6	25.7 ± 8.5	13.8 ± 2.1	6.2 ± 2.6
Age (y)	56.4 ± 7.3	57.0 ± 7.5	56.8 ± 7.6	56.9 ±7.3	56.6 ± 7.5	56.7 ± 7.6
Sex, *n* (%)						
Female	297 (41.1)	349 (49.9)	426 (60.8)	296 (41.0)	351 (50.1)	425 (60.6)
Male	425 (58.9)	351 (50.1)	275 (39.2)	426 (59.0)	349 (49.9)	276 (39.4)
Annual household income, *n* (%)						
<$50,000	223 (30.9)	202 (28.9)	240 (34.2)	217 (30.1)	217 (31.0)	231 (33.0)
$50,000<$100,000	307 (42.5)	297 (42.4)	301 (42.9)	310 (42.9)	286 (40.9)	309 (44.1)
≥$100,000	192 (26.6)	201 (28.7)	160 (22.8)	195 (27.0)	197 (28.1)	161 (23.0)
Smoking status, *n* (%)						
Never	282 (39.1)	285 (40.7)	285 (40.7)	290 (40.2)	275 (39.3)	287 (40.9)
Past	317 (43.9)	332 (47.4)	303 (43.2)	316 (43.8)	327 (46.7)	309 (44.1)
Current	123 (17.0)	83 (11.9)	113 (16.1)	116 (16.1)	98 (14.0)	105 (15.0)
BMI (kg/m^2^)	29.0 ± 5.4	28.4 ± 5.8	28.2 ± 5.1	28.4 ± 5.2	28.8 ± 5.8	28.4 ± 5.3
Physical activity level, *n* (%)						
Low	136 (18.8)	116 (16.6)	103 (14.7)	122 (16.9)	114 (16.3)	119 (17.0)
Moderate	269 (37.3)	263 (37.6)	271 (38.7)	259 (35.9)	288 (41.1)	256 (36.5)
High	317 (43.9)	321 (45.9)	327 (46.7)	341 (47.2)	298 (42.6)	326 (46.5)
Cardiometabolic conditions, *n* (%)						
Hypertension	240 (33.2)	270 (38.6)	279 (39.8)	251 (34.8)	259 (37.0)	279 (39.8)
High blood cholesterol	300 (41.6)	291 (41.6)	274 (39.1)	305 (42.2)	284 (40.6)	276 (39.4)
Both	182 (25.2)	139 (19.9)	148 (21.1)	166 (23.0)	157 (22.4)	146 (20.8)
Cardiopreventive medication use, *n* (%)						
Antihypertensive medication	330 (45.7)	320 (45.7)	325 (46.4)	332 (46.0)	313 (44.7)	330 (47.1)
Antihypertensives (C02)	10 (1.4)	3 (0.4)	8 (1.1)	8 (1.1)	4 (0.6)	9 (1.3)
Diuretics (C03)	59 (8.2)	70 (10.0)	61 (8.7)	63 (8.7)	64 (9.1)	63 (9.0)
Beta blocking agents (C07)	72 (10.0)	73 (10.4)	68 (9.7)	83 (11.5)	59 (8.4)	71 (10.1)
Calcium channel blockers (C08)	69 (9.6)	71 (10.1)	60 (8.6)	67 (9.3)	62 (8.9)	71 (10.1)
Agents acting on the renin-angiotensin system (C09)	228 (31.6)	203 (29.0)	230 (32.8)	224 (31.0)	212 (30.3)	225 (32.1)
Combination therapy for hypertension	96 (13.3)	87 (12.4)	88 (12.6)	100 (13.9)	81 (11.6)	90 (12.8)
Cholesterol-lowering medication	276 (38.2)	210 (30.0)	227 (32.4)	271 (37.5)	224 (32.0)	218 (31.1)
Statin	266 (36.8)	206 (29.4)	224 (32.0)	261 (36.2)	220 (31.4)	215 (30.7)
Ezetimibe	22 (3.1)	11 (1.6)	4 (0.6)	20 (2.8)	12 (1.7)	5 (0.7)
Combination therapy for hypercholesterolemia	12 (1.7)	7 (1.0)	1 (0.1)	10 (1.4)	8 (1.1)	2 (0.3)
Using medication for hypertension or hypercholesterolemia	489 (67.7)	452 (64.6)	468 (66.8)	495 (68.6)	447 (63.9)	467 (66.6)
Using medications for hypertension and hypercholesterolemia	117 (16.2)	78 (11.1)	84 (12.0)	108 (15.0)	90 (12.9)	81 (11.6)
Blood pressure (mmHg)						
Systolic	129 ± 16	129 ± 15	128 ± 16	129 ± 16	129 ± 15	127 ± 16
Diastolic	77 ± 10	76 ± 10	76 ± 10	76 ± 10	77 ± 10	76 ± 10
Plasma lipids (mmol/L)						
Total cholesterol	5.11 ± 1.05	5.34 ± 1.03	5.27 ± 1.03	5.21 ± 1.07	5.27 ± 1.00	5.23 ± 1.05
LDL cholesterol	3.05 ± 0.89	3.20 ± 0.88	3.12 ± 0.90	3.09 ± 0.89	3.16 ± 0.88	3.11 ± 0.90
HDL cholesterol	1.12 ± 0.34	1.23 ± 0.39	1.27 ± 0.40	1.16 ± 0.36	1.21 ± 0.39	1.26 ± 0.40
Framingham risk score, percentage	11.6 ± 3.4	11.1 ± 3.4	10.8 ± 3.5	11.7 ± 3.6	11.2 ± 3.5	10.8 ± 3.5
Dietary intakes						
Alternate healthy eating index, points	46.0 ± 10.1	51.4 ± 10.3	52.5 ± 11.0	46.6 ± 10.2	50.5 ± 10.4	52.7 ± 11.0
Energy intake (kcal/d)	2085 ± 715	1899 ± 647	1722 ± 658	2075 ± 688	1880 ± 684	1752 ± 661
Saturated fats, percentage of calories per day	10.7 ± 2.7	10.7 ± 2.6	10.0 ± 2.9	10.8 ± 2.6	10.6 ± 2.6	10.0 ± 3.0
Unsaturated fats, percentage of calories per day	19.3 ± 4.3	19.7 ± 3.8	19.3 ± 4.8	19.6 ± 3.9	19.7± 4.1	19.1 ± 4.9
Sodium (mg/d)	3032 ± 1174	2794 ± 1089	2540 ± 1062	3093 ± 1179	2756 ± 1089	2517 ± 1033
Sugar, percentage of calories per day	21.9 ± 8.2	22.2 ± 6.8	23.5 ± 7.9	23.7 ± 8.3	21.7 ± 7.2	22.1 ± 7.4
Fiber (g/d)	19.7 ± 8.7	20.3 ± 8.8	20.6 ± 10.3	20.4 ± 8.5	19.8 ± 8.9	20.4 ± 10.4
Alcohol consumption (g/d)	9.6 ± 12.9	11.9 ± 14.2	12.9 ± 26.4	10.2 ± 12.1	12.4 ± 21.5	11.8 ± 21.5

1 Continuous variables are presented as mean ± SD. Categorical variables are presented as count (%).

### Foods of concern and CVD risk

**FIGURE 2:**
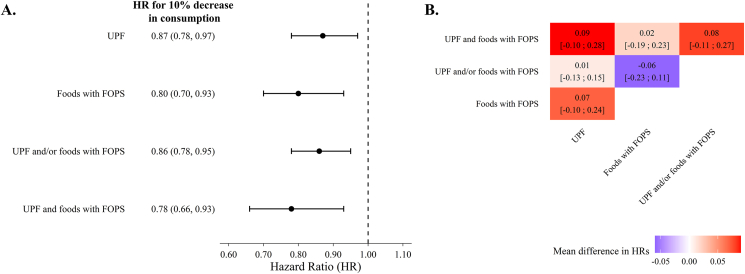
(A) HRs and 95% CIs for cardiovascular disease risk associated with a 10% lower difference in the consumption of foods of concern (as a percentage of grams per day), accompanied by a proportional increase in intake of foods not of concern, based on different modeling approaches to their definition. Models were adjusted for age, sex, household income, smoking status, energy intake, hypertension status, high blood cholesterol status, physical activity level, alcohol consumption, and BMI. (B) Mean differences in HRs for cardiovascular disease risk across different modeling approaches to defining foods of concern. The heatmap shows the mean differences in HRs (95% CI) between all pairs. ∗Two-sided *P* < 0.05 based on a paired *t*-test comparing HRs. CIs, confidence intervals; FOPS, front-of-package nutrition symbol; HRs, hazard ratios; UPF, ultraprocessed foods.

The average follow-up duration was 9.31 (± SD: 1.52) y. A total of 179 CVD events were identified over 19,758 person-years, yielding an incidence rate of 9.06 per 1000 person-years. In the full multivariable-adjusted model (model 3), a 10% lower difference in UPF consumption, and proportional higher difference in intake of non-UPF, was associated with a lower risk of CVDs (HR: 0.87; 95% CI: 0.78, 0.97) (Figure 2A). For foods requiring a FOPS, the corresponding HR was 0.80 (95% CI: 0.70, 0.93). A 10% lower difference in the total consumption of foods that are UPF and/or with FOPS (with and proportional higher difference in intake of non-UPF and/or non-FOPS) was associated with a lower risk of CVDs (HR: 0.86; 95% CI: 0.78, 0.95), as was the intake of foods that are both UPF and with FOPS (HR: 0.78; 95% CI: 0.66, 0.93). E-values for these 4 associations were all >1.50 (i.e., an unmeasured confounder would need to be associated with both the exposure and the outcome by a risk ratio of ≥1.50 each, above and beyond measured covariates, to fully explain away the association; Supplementary Table 5). No evidence of differences in mean HRs was observed between the 4 approaches to foods of concern classification (Figure 2B**).** When the risk of CVDs was assessed per tertiles of consumption of foods of concern, results corroborated those from the continuous analysis (Supplementary Table 5). Supplementary Figure 3 illustrates the survival probability over time across tertiles of consumption of foods of concern.

When the intake was expressed in percentage of calories per day, the direction of the association remained unchanged, but its strength diminished, with the highest bound of the 95% CIs for a 10% lower difference in intake was nearing the threshold of statistical significance (Supplementary Table 6). When foods with unclear level of processing or with uncertain FOPS eligibility were removed, results were unchanged: a 10% lower difference in consumption was associated with lower risk, albeit here again, the strength of the association was weaker (Supplementary Table 7). A 10% lower difference in consumption of foods of concern was also associated with a lower risk of CVDs when the sample was restricted to individuals with hypertension or those with high blood cholesterol (Supplementary Tables 8 and 9).

### Cardiopreventive medication and CVD risk

Characteristics of the 1258 participants with hypertension, according to medication use, are presented in Supplementary Table 10. Those using medication were older, had a higher BMI, and were more likely to have high blood cholesterol and to use cholesterol-lowering medication. Among the 1334 participants with high blood cholesterol, differences between medication users and nonusers were similar to those of participants with medicated or unmedicated hypertension (Supplementary Table 11). No evidence of a lower risk of CVDs was observed among the 975 individuals with medicated hypertension compared with the 283 with unmedicated hypertension (HR: 1.12; 95% CI: 0.72, 1.76) (Supplementary Table 12), albeit systolic and diastolic BPs were lower among individuals using medication (Supplementary Table 13). Cholesterol-lowering medication use was associated with a lower risk of CVDs (HR: 0.59; 95% CI: 0.39, 0.89).

### Interaction between diet and medication

**FIGURE 3:**
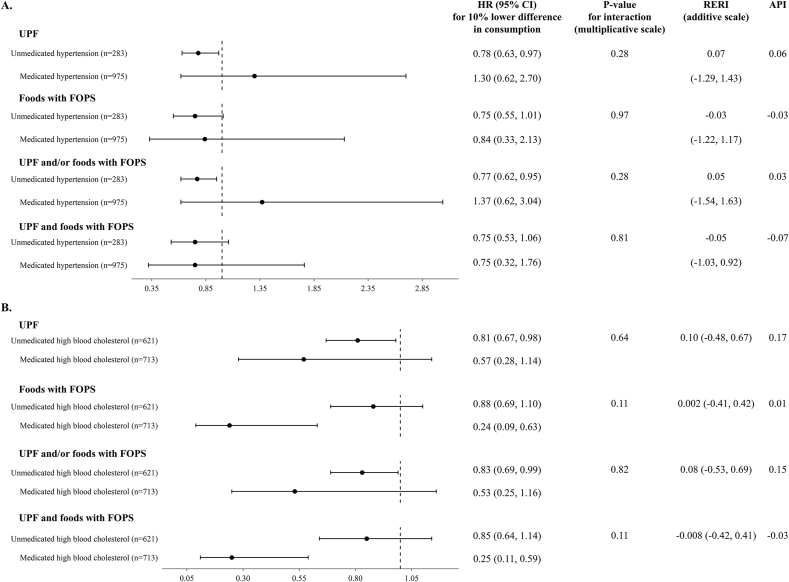
HRs and 95% CIs for cardiovascular disease risk associated with a 10% lower difference in the consumption of foods of concern (as a percentage of grams per day), accompanied by a proportional increase in intake of foods not of concern, based on different modeling approaches to their definition, stratified by medication use among (A) individuals with hypertension (*n* = 1258) and (B) individuals with high blood cholesterol (*n* = 1334). Models were adjusted for age, sex, household income, smoking status, energy intake, alcohol consumption, physical activity level, concomitant high blood cholesterol status or concomitant hypertension status and BMI. The models included an interaction term between medication status and the foods of concern variable. The RERI and the API were calculated from the interaction model. API, attributable proportion due to interaction; CIs, confidence intervals; FOPS, front-of-package nutrition symbol; HRs, hazard ratios; RERI, relative excess risk due to interaction; UPF, ultraprocessed foods.

Finally, we found no evidence of interaction between the consumption of foods of concern (per 10% lower difference in intake, with a proportional increase in intake of foods not of concern) and medication use in relation to CVD risk on the multiplicative scale, with all interaction *P* values > 0.09 (Figure 3). On the additive scale, we similarly found no evidence of departure from additivity: mean RERI values ranged from –0.05 to 0.10, with corresponding 95% CIs overlapping 0, and API values ranged from –0.07 to 0.17.

## Discussion

In this prospective study within the CARTaGENE cohort, among participants with hypertension and/or high blood cholesterol, lower consumption of foods of concern—defined either as UPFs or as foods that would require a FOPS under the 2026 Canadian regulation—was associated with a lower risk of CVDs. There was no evidence that the association with the risk of CVD differed between UPF and foods with FOPS. Additionally, we found no evidence of departure from additivity between BP- or cholesterol-lowering medication use and the consumption of foods of concern in relation with CVD risk. Overall, our study suggests that lower consumption of foods of concern, i.e., UPF and/or foods with FOPS, is linked to a lower risk of CVD in at-risk individuals, independent of concomitant medication use.

Our study primarily demonstrates that lower consumption of foods of concern—whether defined as UPFs and/or foods requiring a FOPS—is associated with a lower risk of CVD among individuals with hypertension and/or high blood cholesterol. Our results on the association between UPF consumption and CVD risk are consistent with previous studies on UPF intake and risk of CVDs conducted in generally healthy individuals [4]. Likewise, our results on foods with FOPS are also consistent with the general consensual understanding on the association between sodium, saturated fat, and sugar consumption and CVD risk [[43], [44], [45]]. Even though we cannot generalize our data to the Canadian adult population, our data support the relevance of the new regulation. This contribution is particularly important, considering a recent analysis that leveraged the Canadian Community Health Survey-Nutrition 2004 and found no evidence of association between the individual intakes of the nutrients targeted by this policy and CVD risk [46]. Notably, about half of the foods classified as UPFs were also subject to FOPS labeling. This highlights 2 key points: *1*) a significant proportion of UPFs contain excessive saturated fat, sodium, or sugars, as previously reported, and *2*) the Nova classification and Canadian FOPS system capture distinct and complementary food characteristics. This distinction is particularly relevant as we found that UPF consumption was associated with CVD risk to the same extent as foods with FOPS. This further supports concerns over the accumulating data showing that, beyond nutritional composition, processing-related factors such as additives, neoformed compounds, and ingredients of rare culinary use may also play a role in CVD development [11]. In that regard, the lack of significant evidence linking UPFs that exclude FOPS-labeled foods to CVD risk found in our sensitivity analyses should be interpreted in the context of their low consumption, which may have limited statistical power. The same applies to the association between the intake of food items with FOPS that were not UPF and CVD risk. Regarding FOPS, high sodium content was the most frequently met criterion per the CDHQII, followed by saturated fat and sugar. Whether this pattern reflects the broader Canadian food environment remains to be determined, but it is nonetheless important for CVD prevention. Our findings suggest that the FOPS policy could reduce CVD risk among at-risk individuals if it effectively lowers the intake of labeled foods and/or nutrients of concern. Although this policy only partially overlaps with the UPF concept, our results do not suggest that the lack of explicit consideration for UPFs undermines its potential impact: targeting UPF consumption remains scientifically valid. Nonetheless, although this was beyond the scope of the present study, accumulating evidence suggests that consumption of certain UPF subgroups may be associated with a lower risk of CVD (e.g., ultraprocessed whole-grain breads) [4], raising important questions about how to design and implement policies targeting UPF. Similar issues could also arise for foods displaying a FOPS; for example, a substantial proportion of whole-grain breads would meet the “high in sodium” threshold under the Canadian regulation [47].

Our interaction analyses showed no evidence of departure from additivity between the use of BP- or cholesterol-lowering medications and the consumption of UPFs or foods with FOPS in relation to CVD risk. The absence of departure from additivity suggests that the association between consumption of foods of concern and CVD risk is neither stronger nor weaker when medication is used concomitantly. This is consistent with analyses restricted to participants with hypertension or hypercholesterolemia, in which lower consumption of foods of concern was associated with lower CVD risk, even after adjustment for medication use. Physiologically, additive effects are plausible because diet can improve pathways and CVD risk factors not directly targeted by medication. For example, statins reduce plasma LDL cholesterol levels by inhibiting intracellular cholesterol synthesis [48]. In parallel, improving diet quality can reduce cholesterol absorption and the production of apolipoprotein B–containing lipoproteins, further lowering LDL cholesterol levels [49,50]. Diet can also confer additional benefits by improving BP or glucose homeostasis. Still, we cannot rule out the possibility that these analyses were underpowered to detect a departure from additivity. We also acknowledge that the wide 95% CIs of the RERIs could be consistent with either a synergistic or antagonistic association.

Our results should be interpreted considering certain limitations and strengths. First, diet was assessed only once, which is suboptimal for drawing long-term inferences about dietary intake, due to both potential misreporting and natural variability over time. Still, because our analyses are based on dietary patterns (i.e., overall presence of foods of concern in the diet), they are less susceptible to within-person variation, which supports the validity of our findings in relation to long-term diet [[51], [52], [53], [54]]. Second, even though the CDHQII has been validated, it remains subject to systematic measurement errors [29]. Third, this FFQ was not specifically designed to capture detailed information on the degree of food processing or FOPS eligibility. This may have introduced misclassification, particularly for UPF, because the FFQ did not distinguish between homemade and industrially manufactured multi-ingredient foods. Accordingly, these items were classified as having an uncertain processing level. Still, in our sample, total UPF consumption accounted for a mean of 41% of daily energy intake, which matches the level of intake reported from the Canadian Community Health Surveys (i.e., 43%) [55]. Fourth, given that the FFQ was not part of the main study data collection, we cannot exclude that those who completed had a superior interest toward diet, and potentially better diet quality than those who did not complete it. However, only 1 of 4 CARTaGENE participants who met our inclusion criteria did not complete the FFQ and could not be included in the study because of that. Fifth, we cannot exclude the presence of changes in medication status and/or pharmacological treatment intensity during follow-up, but the CARTaGENE design impeded our ability to capture these. This could explain why we found no evidence of a protective association of BP-lowering medication use with CVD risk. Likewise, the gap between the assessment of medication use (2009–2010) and the completion of the FFQ (2012) may have caused further confusion. However, the nearly 10-y follow-up is sufficient to study CVD development, especially in a 40–69-y-old cohort. Sixth, we acknowledge that categorizing medication use dichotomously (yes/no) rather than using an intensity-sensitive approach (e.g., based on drug type and/or dosage) is a limitation. However, a more detailed classification would likely have increased the power of our analyses, strengthening the associations with CVD risk or risk factors, thus unlikely to alter our conclusions. In terms of strengths, the linkage with an administrative health database and the use of the same validated event-detection algorithm as the Quebec Institute for Public Health provides high confidence in incident event identification. Finally, the numerous sensitivity analyses conducted to challenge assumptions did not reveal major inconsistencies.

In conclusion, our study suggests that lower consumption of foods of concern, i.e., UPF and/or foods with FOPS, is linked to a lower risk of CVD in at-risk individuals, independent of concomitant medication use.

## Author contributions

The authors’ responsibilities were as follows – J-PD-C: designed the research and had primary responsibility for the final content; LL, J-PD-C: conducted the research and wrote the manuscript; LL, JL-L, J-PD-C: analyzed the data; and all authors: reviewed the manuscript, provided feedback, and read and approved the final manuscript.

## Data availability

Data described in the manuscript will not be made publicly available. Additional information on the procedures for obtaining and accessing data from the CARTaGENE cohort is described at https://cartagene.qc.ca/. Analytic codes are available at https://github.com/Jacob-Lessard-Lord/CaG_FoC_CVD.

## Funding

The study received funding from the Réseau Québécois de Recherche sur les Médicaments (RQRM) and the Réseau de recherche en santé cardiométabolique, diabète et obésité (CMDO). The funders had no role in the design, execution, or interpretation of the research. LL is the recipient of a doctoral’s scholarship from the Fonds d’enseignement et de recherche de la Faculté de pharmacie de l’Université Laval and from the Centre Nutrition Santé et Société of the Institut sur la Nutrition et les Aliments Fonctionnels. JL-L is supported by a Mitacs-Acceleration scholarship and a Canadian Institutes of Health Research (CIHR) Fellowship. J-PD-C is a research scholar of the Fonds de recherche du Québec – Santé.

## Conflict of interest

Authors declare no conflict of interest for the current work. J-PD-C received consulting honoraria and/or investigator-initiated research grants from the Dairy Farmers of Canada and the Weston Family Foundation in the past 5 years, unrelated to this work.
