# Supplementary material for: Foods of concern, cardiopreventive medication use and risk of cardiovascular diseases: a prospective study in the CARTaGENE cohort
Source: Am J Clin Nutr. 2026 Feb 6;123(4):101234. doi: 10.1016/j.ajcnut.2026.101234 (PMC13084580; doi:10.1016/j.ajcnut.2026.101234)
Supplement: Multimedia component 1 [file mmc1.docx]

**Table of contents**

[Supplementary Table S1: Classification of all 285 food items from the Canadian Dietary History Questionnaire II according to whether they are ultra-processed and/or require a front-of-package nutrition symbol. 3](#_Toc217309226)

[Supplementary Table S2: Number (percent) of missing values for each covariable among the 2,123 participants included in the study. 23](#_Toc217309227)

[Supplementary Table S3: Number of included participants with hypertension and/or high blood cholesterol according to medication status.^1^ 24](#_Toc217309228)

[Supplementary Table S4: Characteristics of the 2,123 participants included in the study relative to the 730 that met the inclusion/exclusion criteria but did not complete the FFQ. 25](#_Toc217309229)

[Supplementary Table S5: Hazard ratios (HRs) and 95% confidence intervals (CIs) for cardiovascular disease risk according to tertiles of consumption of foods of concerns (as percentage of grams per day).^1^ 29](#_Toc217309230)

[Supplementary Table S6: Hazard ratios (HRs) and 95% confidence intervals (CIs) for cardiovascular disease risk according to consumption of foods of concerns (as percentage of calories per day).^1^ 33](#_Toc217309231)

[Supplementary Table S7: Hazard ratios (HRs) and 95% confidence intervals (CIs) for cardiovascular disease risk according to consumption of foods of concerns (as percentage of grams per day), using alternative modeling approaches to consumption quantification.^1^ 37](#_Toc217309232)

[Supplementary Table S8: Hazard ratios (HRs) and 95% confidence intervals (CIs) for cardiovascular disease risk according to consumption of foods of concerns (in percentage of grams per day) among individuals with hypertension (n=1,258).^1^ 44](#_Toc217309233)

[Supplementary Table S9: Hazard ratios (HRs) and 95% confidence intervals (CIs) for cardiovascular disease risk according to consumption of foods of concerns (in percentage of grams per day) among individuals with high blood cholesterol (n=1,334).^1^ 48](#_Toc217309234)

[Supplementary Table S10: Characteristics of the 1,258 participants with hypertension according to BP-lowering medication use.^1^ 52](#_Toc217309235)

[Supplementary Table S11: Characteristics of the 1,334 participants with high blood cholesterol according to cholesterol-lowering medication use.^1^ 55](#_Toc217309236)

Supplementary Table S12: Hazard ratios (HRs) and 95% confidence intervals (Cis) for cardiovascular disease risk according to medication status.^1^…………………………………….58

[Supplementary Table S13: Differences in blood pressure and LDL-cholesterol according to medication status at baseline. 59](#_Toc217309237)

[Supplementary Figure S1: Flow-chart of participants’ selection. 61](#_Toc217309238)

[Supplementary Figure S2: Distribution of the 285 food items from the Canadian dietary history questionnaire II across the foods with front-of-package nutrition symbol and ultra-processed foods classifications, excluding food items with uncertain category. 62](#_Toc217309239)

[Supplementary Figure S3: Directed acyclic graph. 63](#_Toc217309240)

[Supplementary Figure S4: Survival curves for the association between consumption of AA) ultra-processed foods (UPF); (B) foods with a front-of-package symbol (FOPS); (C) foods that are ultra-processed or with a FOPS; (D) foods that are both ultra-processed and with a FOPS. 64](#_Toc217309241)

# **Supplementary Table S1: Classification of all 285 food items from the Canadian Dietary History Questionnaire II according to whether they are ultra-processed and/or require a front-of-package nutrition symbol.**

| **Food Items** | **Ultra-processed food** | **Food with front-of-package nutrition symbol** | **Food with front-of-package nutrition symbol - sodium** | **Food with front-of-package nutrition symbol – saturated fat** | **Food with front-of-package nutrition symbol - sugar** |
| --- | --- | --- | --- | --- | --- |
| Apples | N | N |  |  |  |
| Applesauce/cooked apples | N | Y |  |  | X |
| Apricots, dried | N | N |  |  |  |
| Artificial sweeteners | Y | N |  |  |  |
| Artificial sweeteners - Aspartame | Y | N |  |  |  |
| Artificial sweeteners - cyclamate | Y | N |  |  |  |
| Asparagus, no fat added | N | N |  |  |  |
| Avocado, guacamole | N | N |  |  |  |
| Bacon, lean/Canadian | Y | Y | X | X |  |
| Bacon, regular | Y | Y | X | X |  |
| Baked beans | N | U | X |  | X |
| Bananas | N | N |  |  |  |
| Battered/Fish sticks | Y | Y | X |  |  |
| Beans, fat added | N | Y | X |  |  |
| Beans, no fat added | N | N |  |  |  |
| Beef stews/pot pies/mixtures | U | U | X |  |  |
| Beef, burgers, lean | U | U |  | X (exemption if fresh meat) |  |
| Beef, burgers, regular | U | U |  | X (exemption if fresh meat) |  |
| Beef, ground, meatballs/loaf/mixtures | N | N |  |  |  |
| Beef, roast | N | N |  |  |  |
| Beef, steaks, lean | N | N |  |  |  |
| Beef, steaks, regular | N | N |  |  |  |
| Beer | N | N |  |  |  |
| Berries | N | N |  |  |  |
| Biscuits, all | Y | N |  |  |  |
| Bread/rolls whole grains | Y | Y | X |  |  |
| Breads/rolls, white | Y | Y | X |  |  |
| Broccoli, no fat added | N | N |  |  |  |
| Brussel Sprouts, no fat added | N | N |  |  |  |
| Butter, other uses | N | N |  |  |  |
| Butter, other uses, reduced fat | N | N |  |  |  |
| Butter/ reduced fat/ on bread | N | N |  |  |  |
| Butter/ reduced fat/ on pancakes or waffles | N | N |  |  |  |
| Butter/ reduced fat/ on potatoes | N | N |  |  |  |
| Butter/ reduced fat/ on vegetables | N | N |  |  |  |
| Butter/ regular/ on bread | N | N |  |  |  |
| Butter/ regular/ on pancakes or waffles | N | N |  |  |  |
| Butter/ regular/ on potatoes | N | N |  |  |  |
| Butter/ regular/ on vegetables | N | N |  |  |  |
| Cabbage/sauerkraut | N | N |  |  |  |
| Cakes | U | U |  | X | X |
| Candy, chocolate | Y | Y |  | X | X |
| Candy, not chocolate | Y | Y |  |  | X |
| Cantaloupe | N | N |  |  |  |
| Carrots, no fat added | N | N |  |  |  |
| Cauliflower, no fat added | N | N |  |  |  |
| Cheese sauce | N | Y | X | X |  |
| Cheese, low fat | N | N |  |  |  |
| Cheese, regular | N | N |  |  |  |
| Cheeseburger, fast food | Y | Y | X | X |  |
| Cheesecake | Y | Y | X | X | X |
| Chicken, dark no skin | N | N |  |  |  |
| Chicken, dark with skin | N | N |  |  |  |
| Chicken, fried, dark no skin | Y | Y | X | X |  |
| Chicken, fried, dark with skin | Y | Y |  | X |  |
| Chicken, fried, light no skin | Y | N |  |  |  |
| Chicken, fried, light with skin | Y | N |  |  |  |
| Chicken, light no skin | N | N |  |  |  |
| Chicken, light with skin | N | N |  |  |  |
| Chicken, mixtures | U | U | X |  |  |
| Chicken/turkey ground | N | N |  | X (exemption) |  |
| Chili | U | U | X |  |  |
| Chocolate, fudge, butterscotch toppings | Y | Y |  |  | X |
| Chow mein noodles | N | N |  |  |  |
| Cooked spinach/greens, no fat added | N | N |  |  |  |
| Coffee drinks, decaffeinated, cream/sugar | Y | Y |  | X |  |
| Coffee drinks, regular, cream/sugar | Y | Y |  | X |  |
| Coffee, decaffeinated, no cream/sugar | N | N |  |  |  |
| Coffee, regular, no cream/sugar | N | N |  |  |  |
| Cold cuts, low fat | Y | Y | X |  |  |
| Cold cuts, poultry | Y | Y | X |  |  |
| Cold cuts, regular | Y | Y | X | X |  |
| Coleslaw | Y | N |  |  |  |
| Cookies, brownies | Y | N |  |  |  |
| Corn chips | Y | Y | X |  |  |
| Corn, no fat added | N | N |  |  |  |
| Cornbread/muffins | U | N |  |  |  |
| Cottage/ricotta cheese | N | Y | X |  |  |
| Crackers | Y | Y | X |  |  |
| Cream cheese, low fat | Y | Y |  | X |  |
| Cream cheese, regular | Y | Y |  | X |  |
| Cream sub, whipped | Y | Y |  | X |  |
| Cream, regular or 1/2&1/2 in coffee or tea | N | N |  |  |  |
| Cream, regular, whipped | U | U |  | X |  |
| Crisps/cobblers | Y | Y |  |  | X |
| Croissants | Y | Y |  | X |  |
| Dark chocolate | Y | Y |  | X | X |
| Donuts, sweat rolls, danish, pop tarts | Y | N |  |  |  |
| Dried fruit, no apricots | N | N |  |  |  |
| Dried oregano, rosemary, thyme | N | N |  |  |  |
| Egg rolls, all | Y | Y | X |  |  |
| Eggs, regular | N | N |  |  |  |
| Eggs, salad | Y | N |  |  |  |
| Eggs/whites, substitutes | U | N |  |  |  |
| Energy drink, regular, with caffeine | Y | Y |  |  | X |
| Eng muffin/bagel, white | Y | N |  |  |  |
| Eng muffin/bagel, whole grain | Y | N |  |  |  |
| Fish, not fried, no fat added | N | N |  |  |  |
| Flaxseeds | N | N |  |  |  |
| Fresh basil, cilantro, parsley | N | N |  |  |  |
| Frozen yogurt, ices, sorbet | Y | Y |  |  | X |
| Fruit drinks, diet | Y | N |  |  |  |
| Fruit drinks, regular | Y | Y |  |  | X |
| Fruit salads/other fruits | N | N |  |  |  |
| Game foods | N | N |  |  |  |
| Gelatins | Y | N |  |  |  |
| Granola bars | Y | N |  |  |  |
| Grapefruit, all | N | N |  |  |  |
| Grapes, all | N | N |  |  |  |
| Gravy | Y | N |  |  |  |
| Ham, cold cut/ lunch meat, low fat | Y | Y | X | X |  |
| Ham, cold cut/ lunch meat, regular | Y | Y | X | X |  |
| Ham, not luncheon | N | N | X (exemption) |  |  |
| Hamburger, fast food | Y | Y | X | X |  |
| Hot breakfast cereals, fat added | N | N |  |  |  |
| Hot breakfast cereals, no fat added | N | N |  |  |  |
| Hot dogs, regular | Y | Y | X | X |  |
| Hot dogs, turky/low fat | Y | Y | X | X |  |
| Hot/cold herbal tea | N | N |  |  |  |
| Ice cream, regular | Y | Y |  | X | X |
| Ice cream/ice milk, low fat | Y | Y |  |  | X |
| Jams, jelly, regular | Y | Y |  |  | X |
| Lard, fatback, bacon fat | N | Y |  | X |  |
| Lasagna, raviolis, shells | U | U | X | X |  |
| Lettuce, not dark green | N | N |  |  |  |
| Lettuce/ dark green | N | N |  |  |  |
| Liver, liverwurst | N | Y | X | X |  |
| Macaroni and cheese | Y | Y | X | X |  |
| Mangos | N | N |  |  |  |
| Maple syrup on pancakes | N | N |  |  |  |
| Margarine, other uses, diet | Y | N |  |  |  |
| Margarine, other uses, regular | Y | N |  |  |  |
| Margarine/ diet/ on bread | Y | N |  |  |  |
| Margarine/ diet/ on pancakes or waffle | Y | N |  |  |  |
| Margarine/ diet/ on potatoes | Y | N |  |  |  |
| Margarine/ diet/ on vegetables | Y | N |  |  |  |
| Margarine/ regular/ on bread | Y | N |  |  |  |
| Margarine/ regular/ on pancakes or waffles | Y | N |  |  |  |
| Margarine/ regular/ on potatoes | Y | N |  |  |  |
| Margarine/ regular/ on vegetables | Y | N |  |  |  |
| Mayonnaise, diet on salad | Y | N |  |  |  |
| Mayonnaise, regular on salad | Y | N |  |  |  |
| Mayonnaise/ diet on sandwich | Y | N |  |  |  |
| Mayonnaise/ regular on sandwich | Y | N |  |  |  |
| Meal replacement, liquid | Y | N |  |  |  |
| Meal replacement bar | Y | N |  |  |  |
| Meat based snacks (jerky) | Y | Y | X | X |  |
| Mexican mixtures, all | Y | Y |  | X |  |
| Milk evaporated/condensed, in coffee/tea | Y | N |  |  |  |
| Milk, 1% not in coffee/tea | N | N |  |  |  |
| Milk, 1%, in cereals | N | N |  |  |  |
| Milk, 1%, in coffee or tea | N | N |  |  |  |
| Milk, 2% not in coffee/tea | N | N |  |  |  |
| Milk, 2%, in cereals | N | N |  |  |  |
| Milk, 2%, in coffee or tea | N | N |  |  |  |
| Milk, almond, in cereals | Y | N |  |  |  |
| Milk, almond, in coffee or tea | Y | N |  |  |  |
| Milk, almond, not in coffee/tea | Y | N |  |  |  |
| Milk, nonfat/skim not in coffee/tea | N | N |  |  |  |
| Milk, nonfat/skim, in cereals | N | N |  |  |  |
| Milk, nonfat/skim, in coffee or tea | N | N |  |  |  |
| Milk, rice, in cereals | N | N |  |  |  |
| Milk, rice, in coffee or tea | N | N |  |  |  |
| Milk, rice, not in coffee/tea | N | N |  |  |  |
| Milk, soy, in cereals | N | N |  |  |  |
| Milk, soy, in coffee or tea | N | N |  |  |  |
| Milk, soy, not in coffee/tea | N | N |  |  |  |
| Milk, whole not in coffee/tea | N | N |  |  |  |
| Milk, whole, in cereals | N | N |  |  |  |
| Milk, whole, in coffee or tea | N | N |  |  |  |
| Milk/ chocolate, reduced fat to drink | Y | Y |  |  | X |
| Milk/ chocolate, whole to drink | Y | Y |  | X | X |
| Milkshakes/sodas | Y | Y |  | X | X |
| Muffins/dessert breads | U | U | X |  | X |
| Non-dairy cream, liquid, diet in coffee or tea | Y | N |  |  |  |
| Non-dairy cream, liquid, regular in coffee or tea | Y | N |  |  |  |
| Non-dairy cream, powdered, diet in coffee or tea | Y | N |  |  |  |
| Non-dairy cream, powdered, regular in coffee or tea | Y | N |  |  |  |
| Nuts, whole | N | N |  |  |  |
| Nuts/seeds, butters | N | N |  |  |  |
| Oil sprays, Pam | Y | N |  |  |  |
| Oils, canola | N | N |  |  |  |
| Oils, corn | N | N |  |  |  |
| Oils, olive | N | N |  |  |  |
| Oils, other | N | N |  |  |  |
| Olives, all | N | Y | X |  |  |
| Onions, no fat added | N | N |  |  |  |
| Orange/grapefruit juice, all | N | Y |  |  | X |
| Orange/grapefruit juice, calcium fortified | N | Y |  |  | X |
| Oranges, tangelo etc | N | N |  |  |  |
| Other juice | N | Y |  |  | X |
| Other melon | N | N |  |  |  |
| Other seeds | N | N |  |  |  |
| Other vegetables, no fat added | N | N |  |  |  |
| Pancake, waffle, French toast | U | U | X |  |  |
| Pasta salad | U | U | X |  |  |
| Pasta, fat added | N | N |  |  |  |
| Pasta, meat/fish sauce | U | U | X |  |  |
| Pasta, meatless red sauce | U | N |  |  |  |
| Pasta, no fat added | N | N |  |  |  |
| Peaches/nectarines/plums | N | N |  |  |  |
| Peanuts | N | N |  |  |  |
| Pears | N | N |  |  |  |
| Peas, no fat added | N | N |  |  |  |
| Peppers, hot | N | N |  |  |  |
| Peppers, no fat added | N | N |  |  |  |
| Pickled vegetables/fruits | N | Y | X |  |  |
| Pies, cream/custard/other | Y | Y |  | X | X |
| Pies, fruit | Y | Y |  | X | X |
| Pies, pecan | Y | Y |  | X |  |
| Pies, pumpkin /sweat potato, etc | Y | Y |  | X |  |
| Pineapple | N | N |  |  |  |
| Pizza, with meat | Y | Y | X |  |  |
| Pizza, without meat | Y | Y | X |  |  |
| Plantains | N | N |  |  |  |
| Popcorn | Y | Y | X |  |  |
| Pork | N | N |  |  |  |
| Pork, neck, feet, etc | N | N |  |  |  |
| Potato chips | Y | N |  |  |  |
| Potato salads | U | U | X |  |  |
| Potatoes, fried | N | N |  |  |  |
| Potatoes, white, no fat added | N | N |  |  |  |
| Poutine | Y | Y | X | X |  |
| Pretzels, all | Y | Y | X |  |  |
| Puddings/custards | Y | Y |  |  | X |
| Raw spinach/greens | N | N |  |  |  |
| Red wine | N | N |  |  |  |
| Rice/grains, fat added | N | U | X |  |  |
| Rice/grains, no fat added | N | N |  |  |  |
| Roast beef in sandwich | Y | N |  |  |  |
| RTE cereal, good fiber | Y | N |  |  |  |
| RTE cereal, high fiber | Y | Y | X |  |  |
| RTE cereal, highly fortified | Y | Y | X |  |  |
| RTE cereal, other | Y | N |  |  |  |
| Saccharin | Y | N |  |  |  |
| Saccharin (stevia) | Y | N |  |  |  |
| Salad dressing, low fat/fat free on salad or vegetables | Y | Y | X |  |  |
| Salad dressing, regular on salad or vegetables | Y | Y | X |  |  |
| Salmon, fresh tuna, trout | N | N |  |  |  |
| Sausage, regular | Y | Y | X | X |  |
| Sausage, turkey/low fat | Y | Y |  |  | X |
| Shellfish, fat added | N | Y |  |  | X |
| Shellfish, not fried, no fat added | N | N |  |  |  |
| Shortribs/spareribs | N | N |  |  |  |
| Soft drinks, diet, caffeine | Y | N |  |  |  |
| Soft drinks, diet, decaffeinated | Y | N |  |  |  |
| Soft drinks, regular, caffeine | Y | Y |  |  | X |
| Soft drinks, regular, decaffeinated | Y | Y |  |  | X |
| Soups, bean-type | U | U | X |  |  |
| Soups, broth with noodles/rice | U | U | X |  |  |
| Soups, creamed | U | U | X |  |  |
| Soups, with veggies | U | U | X |  |  |
| Sour cream, low fat | N | N |  |  |  |
| Sour cream, regular | N | Y |  | X |  |
| Soy burger or meat substitute | Y | Y | X |  |  |
| Splenda | Y | N |  |  |  |
| Sports drinks | Y | Y |  |  | X |
| Strawberries | N | N |  |  |  |
| String beans, no fat added | N | N |  |  |  |
| Stuffing/dumplings, all | Y | N |  |  |  |
| Sugars/honey, all in coffee or tea | N | N |  |  |  |
| Sushi | N | N |  |  |  |
| Sweet potatoes, no fat added | N | N |  |  |  |
| Tea, decaffeinated, no cream/sugar | N | N |  |  |  |
| Tea, regular, no cream/sugar | N | N |  |  |  |
| Tofu | N | N |  |  |  |
| Tomato catsup | Y | N |  |  |  |
| Tomato juice | N | Y | X |  |  |
| Tomato salsa | Y | N |  |  |  |
| Tomatoes, raw | N | N |  |  |  |
| Tuna canned, oil pack | N | N |  |  |  |
| Tuna canned, water pack | N | N |  |  |  |
| Turkey | N | N |  |  |  |
| Veal, venison, lamb dishes | N | U | X | X |  |
| Vegetable mix, no fat added | N | N |  |  |  |
| Vegetable juice | N | Y | X |  |  |
| Water bottled, fortified | N | N |  |  |  |
| Water, bottled, sweetened | Y | N |  |  |  |
| Water, bottled, unsweet | N | N |  |  |  |
| Water, tap | N | N |  |  |  |
| White sauce | U | N |  |  |  |
| Wine, not red | N | N |  |  |  |
| Winter squash, no fat added | N | N |  |  |  |
| Yogurt/ whole milk | N | N |  |  |  |
| Yogurt/lowfat/nonfat | Y | Y |  |  | X |

# **Supplementary Table S2: Number (percent) of missing values for each covariable among the 2,123 participants included in the study.**

| **Covariable** | **Missing data** |
| --- | --- |
| Age | 0 (0%) |
| Sex | 0 (0%) |
| Household income | 95 (4%) |
| Smoking status | 9 |
| Alcohol consumption | 0 (0%) |
| Physical activity level | 0 (0%) |
| Energy intake | 0 (0%) |
| Body mass index | 13 |

# **Supplementary Table S3: Number of included participants with hypertension and/or high blood cholesterol according to medication status.^1^**

|  | No hypertension | Hypertension, without BP-lowering medication | Hypertension with BP-lowering medication | Total (row) |
| --- | --- | --- | --- | --- |
| No high blood cholesterol | 0 | 203 | 586 | 789 |
| High blood cholesterol,  without cholesterol-lowering medication | 460 | 51 | 110 | 621 |
| High blood cholesterol,  with cholesterol-lowering medication | 405 | 29 | 279 | 713 |
| Total (column) | 865 | 283 | 975 | 2123 |

^1^ A total of 1,334 participants self-reported having high blood cholesterol. A total of 1,258 participants self-reported having hypertension. Abbreviation: BP, blood pressure.

# **Supplementary Table S4: Characteristics of the 2,123 participants included in the study relative to the 730 that met the inclusion/exclusion criteria but did not complete the FFQ.**

| **Characteristics** | **Included participants**  **(n=2,123)** | **All participants meeting the inclusion/exclusion criteria, independent of FFQ completion**  **(n=2,853)** | **Participants meeting the inclusion/exclusion criteria but who did not complete the FFQ**  **(n=730)** |
| --- | --- | --- | --- |
| Age, years | 56.7 ± 7.5 | 56.2 ± 4.6 | 54.7 ± 8.0 |
| Sex, n (%) |  |  |  |
| Female | 1,072 (50.5) | 1,382 (48.4) | 310 (42.5) |
| Male | 1,051 (49.5) | 1,471 (51.6) | 420 (57.5) |
| Annual household income, n (%) |  |  |  |
| <$50,000 | 665 (31.3) | 1,043 (36.6) | 283 (38.8) |
| $50,000<$100,000 | 905 (42.6) | 1,047 (36.7) | 237 (32.5) |
| ≥$100,000 | 553 (26.1) | 763 (26.7) | 210 (28.8) |
| Smoking status, n (%) |  |  |  |
| Never | 852 (40.1) | 1,152 (40.4) | 300 (41.1) |
| Past | 952 (44.8) | 1,258 (44.1) | 306 (41.9) |
| Current | 319 (15.0) | 443 (15.5) | 124 (17.0) |
| Body mass index, kg/m² | 28.5 ± 5.5 | 28.6 ± 5.5 | 28.9 ± 5.4 |
| Physical activity level, n (%) |  |  |  |
| Low | 355 (16.7) | 475 (16.7) | 120 (16.4) |
| Moderate | 803 (37.8) | 1,072 (37.6) | 269 (36.9) |
| High | 965 (45.5) | 1,306 (45.8) | 341 (46.7) |
| Cardiometabolic conditions |  |  |  |
| Hypertension | 789 (37.2) | 1,043 (36.6) | 254 (34.8) |
| High blood cholesterol | 865 (40.7) | 1,169 (41.0) | 304 (41.6) |
| Both | 469 (22.1) | 641 (22.5) | 172 (23.6) |
| Cardiopreventive medication use, n (%) |  |  |  |
| Antihypertensive medication | 975 (45.9) | 1,294 (45.4) | 319 (43.7) |
| Antihypertensives (C02) | 21 (1.0) | 25 (0.9) | 4 (0.5) |
| Diuretics (C03) | 190 (8.9) | 247 (8.7) | 57 (7.8) |
| Beta blocking agents (C07) | 213 (10.0) | 273 (9.6) | 60 (8.2) |
| Calcium channel blockers (C08) | 200 (9.4) | 273 (9.6) | 73 (10.0) |
| Agents acting on the renin-angiotensin system (C09) | 661 (31.1) | 887 (31.1) | 226 (31.0) |
| Combination therapy for hypertension | 271 (12.8) | 365 (12.8) | 94 (12.9) |
| Cholesterol-lowering medication | 713 (33.6) | 951 (33.3) | 238 (32.6) |
| Statin (C10AA) | 696 (32.8) | 934 (32.7) | 238 (32.6) |
| Ezetimibe (C10AX09) | 37 (1.7) | 42 (1.5) | 5 (0.7) |
| Combination therapy for hypercholesterolemia | 20 (0.9) | 25 (0.9) | 5 (0.7) |
| Using at least 1 medication for hypertension or hypercholesterolemia | 1,409 (66.4) | 1,863 (65.3) | 454 (62.2) |
| Using at least 1 medication for hypertension and hypercholesterolemia | 279 (13.1) | 382 (13.4) | 103 (14.1) |
| Blood pressure, mm Hg |  |  |  |
| Systolic | 129 ± 16 | 128 ± 15 | 128 ± 14 |
| Diastolic | 76 ± 10 | 76 ± 10 | 76 ± 10 |
| Plasma lipids, mmol/L |  |  |  |
| Total-cholesterol | 5.24 ± 1.04 | 5.22 ± 1.05 | 5.16 ± 1.08 |
| Triglycerides | 2.06 ± 1.47 | 2.07 ± 1.45 | 2.11 ± 1.40 |
| LDL-cholesterol | 3.12 ± 0.89 | 3.10 ± 0.89 | 3.06 ± 0.90 |
| HDL-cholesterol | 1.21 ± 0.38 | 1.20 ± 0.39 | 1.16 ± 0.39 |
| Framingham risk score, percentage | 11.2 ± 3.4 | 11.1 ± 3.5 | 10.8 ± 3.5 |

Abbreviation: FFQ, food frequency questionnaire.

# **Supplementary Table S5: Hazard ratios (HRs) and 95% confidence intervals (CIs) for cardiovascular disease risk according to tertiles of consumption of foods of concerns (as percentage of grams per day).^1^**

| **Model** | **Tertile 3** | **Tertile 2** | **Tertile 1** | ***P-*value for trend^2^** | **HR (95% CI) for 10% lower difference in consumption** | ***E*-value^3^** |
| --- | --- | --- | --- | --- | --- | --- |
| Ultra-processed foods |  |  |  |  |  |  |
| Mean consumption ± SD | 27.4 ± 12.5 | 12.4 ± 2.0 | 5.5 ± 2.3 |  |  |  |
| Cases/person-years, n | 78/6,610 | 55/6,586 | 46/6,563 |  |  |  |
| Model 1 | 1.00  (reference) | 0.75  (0.52, 1.07) | 0.66  (0.46, 0.96) | 0.01 | 0.88  (0.79, 0.97) | 1.53 |
| Model 2 | 1.00  (reference) | 0.75  (0.52, 1.07) | 0.66  (0.45, 0.96) | 0.02 | 0.88  (0.79, 0.98) | 1.53 |
| Model 3 | 1.00  (reference) | 0.74  (0.52, 1.06) | 0.66  (0.45, 0.96) | 0.01 | 0.87  (0.78, 0.97) | 1.56 |
| Foods with front-of-package symbol |  |  |  |  |  |  |
| Mean consumption ± SD | 25.7 ± 8.5 | 13.8 ± 2.1 | 6.2 ± 2.6 |  |  |  |
| Cases/person-years, n | 79/6,646 | 56/6,530 | 44/6,582 |  |  |  |
| Model 1 | 1.00  (reference) | 0.75  (0.53, 1.05) | 0.62  (0.43, 0.90) | 0.001 | 0.80  (0.70, 0.92) | 1.81 |
| Model 2 | 1.00  (reference) | 0.75  (0.53, 1.06) | 0.62  (0.42, 0.92) | 0.002 | 0.80  (0.69, 0.92) | 1.81 |
| Model 3 | 1.00  (reference) | 0.75  (0.53, 1.06) | 0.63  (0.43, 0.92) | 0.003 | 0.80  (0.70, 0.93) | 1.81 |
| Foods that are ultra-processed and/or have a front-of-package symbol |  |  |  |  |  |  |
| Mean consumption ± SD | 34.5 ± 12.1 | 18.0 ± 2.6 | 8.2 ± 3.4 |  |  |  |
| Cases/person-years, n | 80/6,622 | 55/6,564 | 44/6,573 |  |  |  |
| Model 1 | 1.00  (reference) | 0.70  (0.49, 1.00) | 0.62  (0.43, 0.91) | 0.004 | 0.87  (0.79, 0.96) | 1.56 |
| Model 2 | 1.00  (reference) | 0.70  (0.49, 1.00) | 0.62  (0.42, 0.92) | 0.006 | 0.87  (0.78, 0.96) | 1.56 |
| Model 3 | 1.00  (reference) | 0.69  (0.48, 0.98) | 0.61  (0.41, 0.91) | 0.004 | 0.86  (0.78, 0.95) | 1.60 |
| Foods that are both ultra-processed and with front-of-package symbol |  |  |  |  |  |  |
| Mean consumption ± SD | 17.8 ± 8.1 | 8.8 ± 1.3 | 3.8 ± 1.6 |  |  |  |
| Cases/person-years | 75/6,640 | 54/6,601 | 50/6,518 |  |  |  |
| Model 1 | 1.00  (reference) | 0.78  (0.54, 1.10) | 0.76  (0.53, 1.09) | 0.002 | 0.78  (0.66, 0.92) | 1.88 |
| Model 2 | 1.00  (reference) | 0.78  (0.55, 1.12) | 0.76  (0.53, 1.11) | 0.003 | 0.78  (0.66, 0.92) | 1.88 |
| Model 3 | 1.00  (reference) | 0.78  (0.55, 1.12) | 0.77  (0.53, 1.11) | 0.004 | 0.78  (0.68, 0.93) | 1.88 |

^1^ Model 1 was adjusted for age (years), sex (female, male), smoking status (never, past, current), household income (<$50,000; $50,000–$100,000; >$100,000), alcohol intake (g/day), physical activity level (low, moderate, high), hypertension status (none, unmedicated, medicated), and high blood cholesterol status (none, unmedicated, medicated). Model 2 was additionally adjusted for energy intake (kcal/day). Model 3 was additionally adjusted for energy intake (kcal/day) and body mass index (BMI; kg/m²).

^2^ E-values were calculated using HR (95% CI) for 10% lower difference in consumption.

^3^ *P*-values for trend were calculated by modeling the consumption of foods of concern as a continuous variable.

Abbreviations: CI, confidence interval; HR, hazard ratio; SD, standard deviation.

# **Supplementary Table S6: Hazard ratios (HRs) and 95% confidence intervals (CIs) for cardiovascular disease risk according to consumption of foods of concerns (as percentage of calories per day).^1^**

|  | Tertile 3 | Tertile 2 | Tertile 1 | *P*-value for trend^2^ | HR (95% CI) for 10% lower difference in consumption | *E*-value^3^ |
| --- | --- | --- | --- | --- | --- | --- |
| Ultra-processed foods |  |  |  |  |  |  |
| Mean consumption ± SD | 53.4 ± 7.3 | 40.7 ± 2.6 | 28.2 ± 6.1 |  |  |  |
| Cases/person-years | 64/6,632 | 64/6,544 | 51/6,582 |  |  |  |
| Model 1 | 1.00  (reference) | 1.01  (0.71, 1.45) | 0.85  (0.57, 1.26) | 0.21 | 0.92  (0.80, 1.05) | 1.39 |
| Model 2 | 1.00  (reference) | 1.02  (0.71, 1.46) | 0.86  (0.54, 1.28) | 0.23 | 0.92  (0.80, 1.06) | 1.39 |
| Model 3 | 1.00  (reference) | 1.01  (0.71, 1.45) | 0.84  (0.56, 1.26) | 0.20 | 0.91  (0.80, 1.05) | 1.43 |
| Foods with front-of-package symbol |  |  |  |  |  |  |
| Mean consumption ± SD | 48.5 ± 6.0 | 37.6 ± 2.3 | 26.3 ± 5.7 |  |  |  |
| Cases/person-years | 66/6,645 | 63/6,549 | 50/6,565 |  |  |  |
| Model 1 | 1.00  (reference) | 0.98  (0.69, 1.39) | 0.79  (0.54, 1.17) | 0.19 | 0.90  (0.78, 1.05) | 1.46 |
| Model 2 | 1.00  (reference) | 0.98  (0.69, 1.38) | 0.80  (0.54, 1.18) | 0.21 | 0.91  (0.78, 1.06) | 1.43 |
| Model 3 | 1.00  (reference) | 0.97  (0.69, 1.38) | 0.79  (0.54, 1.17) | 0.19 | 0.90  (0.78, 1.05) | 1.46 |
| Foods that are ultra-processed and/or have a front-of-package symbol |  |  |  |  |  |  |
| Mean consumption ± SD | 59.8 ± 6.5 | 47.7 ± 2.5 | 35.2 ± 6.6 |  |  |  |
| Cases/person-years | 67/6,625 | 60/6,552 | 52/6,582 |  |  |  |
| Model 1 | 1.00  (reference) | 0.93  (0.65, 1.32) | 0.82  (0.55, 1.20) | 0.15 | 0.90  (0.78, 1.04) | 1.46 |
| Model 2 | 1.00  (reference) | 0.93  (0.65, 1.32) | 0.83  (0.56, 1.22) | 0.16 | 0.90  (0.78, 1.04) | 1.46 |
| Model 3 | 1.00  (reference) | 0.93  (0.65, 1.32) | 0.81  (0.55, 1.20) | 0.14 | 0.90  (0.78, 1.04) | 1.46 |
| Foods that are both ultra-processed and with front-of-package symbol |  |  |  |  |  |  |
| Mean consumption ± SD | 41.4 ± 6.6 | 30.5 ± 2.3 | 20.1± 4.9 |  |  |  |
| Cases/person-years | 66/6,672 | 59/6,546 | 54/6,540 |  |  |  |
| Model 1 | 1.00  (reference) | 0.93  (0.65, 1.32) | 0.88  (0.60, 1.28) | 0.24 | 0.91  (0.79, 1.06) | 1.43 |
| Model 2 | 1.00  (reference) | 0.93  (0.65, 1.33) | 0.88  (0.60, 1.29) | 0.26 | 0.92  (0.79, 1.07) | 1.393 |
| Model 3 | 1.00  (reference) | 0.92  (0.64, 1.32) | 0.87  (0.60, 1.27) | 0.23 | 0.91  (0.78, 1.06) | 1.43 |

^1^ Model 1 was adjusted for age (years), sex (female, male), smoking status (never, past, current), household income (<$50,000; $50,000–$100,000; >$100,000), alcohol intake (g/day), physical activity level (low, moderate, high), hypertension status (none, unmedicated, medicated), and high blood cholesterol status (none, unmedicated, medicated). Model 2 was additionally adjusted for energy intake (kcal/day). Model 3 was additionally adjusted for energy intake (kcal/day) and body mass index (BMI; kg/m²).

^2^ *P*-values for trend were calculated by modeling the consumption of foods of concern as a continuous variable.

^3^ E-values were calculated using HRs for each 10% lower difference in intake.

Abbreviations: CI, confidence interval; HR, hazard ratio; SD, standard deviation.

# **Supplementary Table S7: Hazard ratios (HRs) and 95% confidence intervals (CIs) for cardiovascular disease risk according to consumption of foods of concerns (as percentage of grams per day), using alternative modeling approaches to consumption quantification.^1^**

|  | Tertile 3 | Tertile 2 | Tertile 1 | *P*-value for trend^2^ | HR (95% CI) for 10% lower difference in consumption | *E*-value^3^ |
| --- | --- | --- | --- | --- | --- | --- |
| Ultra-processed foods excluding food items with uncertain processing level |  |  |  |  |  |  |
| Mean intake ± SD | 22.4 ± 12.7 | 8.2 ± 1.5 | 3.4 ± 1.5 |  |  |  |
| Cases/person-years, n | 80/6,604 | 49/6,604 | 50/6,551 |  |  |  |
| Model 1 | 1.00 (reference) | 0.63  (0.44, 0.91) | 0.71  (0.50, 1.02) | 0.02 | 0.88  (0.79, 0.98) | 1.53 |
| Model 2 | 1.00 (reference) | 0.63  (0.44, 0.91) | 0.72  (0.50, 1.04) | 0.03 | 0.89  (0.79, 0.99) | 1.50 |
| Model 3 | 1.00 (reference) | 0.62  (0.43, 0.89) | 0.71  (0.50, 1.02) | 0.02 | 0.88  (0.79, 0.98) | 1.53 |
| Foods with front-of-package symbol excluding food items with uncertain symbol eligibility |  |  |  |  |  |  |
| Mean intake ± SD | 19.7 ± 8.6 | 9.0 ± 1.6 | 3.7 ± 1.6 |  |  |  |
| Cases/person-years, n | 79/6,618 | 57/6,561 | 43/6,579 |  |  |  |
| Model 1 | 1.00 (reference) | 0.76  (0.54, 1.08) | 0.59  (0.41, 0.86) | 0.002 | 0.80  (0.69, 0.92) | 1.81 |
| Model 2 | 1.00 (reference) | 0.76  (0.54, 1.08) | 0.60  (0.41, 0.88) | 0.003 | 0.80  (0.69, 0.92) | 1.81 |
| Model 3 | 1.00 (reference) | 0.77  (0.55, 1.09) | 0.60  (0.41, 0.89) | 0.003 | 0.80  (0.69, 0.93) | 1.81 |
| Foods that are ultra-processed and/or have a front-of-package symbol, excluding food items with uncertain processing level or symbol eligibility |  |  |  |  |  |  |
| Mean intake ± SD | 28.5 ± 12.3 | 12.7 ±2.3 | 5.4 ± 2.3 |  |  |  |
| Cases/person-years | 81/6,604 | 57/6,555 | 41/6,600 |  |  |  |
| Model 1 | 1.00  (reference) | 0.71  (0.50, 1.00) | 0.56  (0.38, 0.82) | 0.007 | 0.87  (0.79, 0.96) | 1.56 |
| Model 2 | 1.00  (reference) | 0.71  (0.50, 1.00) | 0.56  (0.38, 0.83) | 0.01 | 0.87  (0.79, 0.97) | 1.56 |
| Model 3 | 1.00  (reference) | 0.70  (0.50, 1.00) | 0.56  (0.38, 0.82) | 0.006 | 0.86  (0.78, 0.96) | 1.60 |
| Ultra-processed foods excluding foods with front-of-package symbol |  |  |  |  |  |  |
| Mean ± SD | 16.9 ± 11.5 | 6.1 ± 1.1 | 2.5 ± 1.1 |  |  |  |
| Cases/person-years | 70/6,660 | 55/6,579 | 54/6,520 |  |  |  |
| Model 1 | 1.00  (reference) | 0.82  (0.57, 1.16) | 0.87  (0.61, 1.25) | 0.23 | 0.93  (0.82, 1.05) | 1.36 |
| Model 2 | 1.00  (reference) | 0.83  (0.58, 1.18) | 0.88  (0.61, 1.28) | 0.23 | 0.93  (0.82, 1.06) | 1.36 |
| Model 3 | 1.00  (reference) | 0.81  (0.57, 1.16) | 0.87  (0.60, 1.26) | 0.14 | 0.92  (0.81, 1.04) | 1.39 |
| Ultra-processed foods excluding food items with uncertain processing level and excluding foods with front-of-package symbol |  |  |  |  |  |  |
| Mean ± SD | 11.6 ± 11.9 | 2.0 ± 0.6 | 0.6 ± 0.3 |  |  |  |
| Cases/person-years | 71/6,629 | 60/6,561 | 48/6,569 |  |  |  |
| Model 1 | 1.00  (reference) | 0.8  (0.62, 1.23) | 0.79  (0.54, 1.15) | 0.47 | 0.95  (0.83, 1.09) | 1.29 |
| Model 2 | 1.00  (reference) | 0.88  (0.62, 1.24) | 0.80  (0.55, 1.14) | 0.52 | 0.95  (0.83, 1.10) | 1.29 |
| Model 3 | 1.00  (reference) | 0.85  (0.60, 1.21) | 0.77  (0.53, 1.13) | 0.37 | 0.94  (0.82, 1.08) | 1.32 |
| Foods with front-of-package symbol excluding ultra-processed foods |  |  |  |  |  |  |
| Mean ± SD | 15.2 ± 5.1 | 7.6 ± 1.4 | 3.0 ± 1.3 |  |  |  |
| Cases/person-years | 70/6,673 | 64/6548 | 45/6538 |  |  |  |
| Model 1 | 1.00  (reference) | 0.91  (0.65, 1.28) | 0.70  (0.48, 1.02) | 0.03 | 0.79  (0.64, 0.98) | 1.88 |
| Model 2 | 1.00  (reference) | 0.92  (0.66, 1.29) | 0.70  (0.48, 1.04) | 0.05 | 0.80  (0.64, 1.00) | 1.81 |
| Model 3 | 1.00  (reference) | 0.93  (0.66, 1.30) | 0.71  (0.48, 1.04) | 0.05 | 0.80  (0.64, 1.00) | 1.81 |
| Foods with front-of-package symbol excluding food items with uncertain symbol eligibility and excluding ultra-processed foods |  |  |  |  |  |  |
| Mean ± SD | 9.0 ± 4.8 | 2.8 ± 0.9 | 0.7 ± 0.4 |  |  |  |
| Cases/person-years | 73/6,666 | 52/6,592 | 54/6,501 |  |  |  |
| Model 1 | 1.00  (reference) | 0.74  (0.52, 1.06) | 0.79  (0.56, 1.14) | 0.10 | 0.81  (0.63, 1.04) | 1.77 |
| Model 2 | 1.00  (reference) | 0.75  (0.53, 1.07) | 0.80  (0.56, 1.15) | 0.11 | 0.82  (0.63, 1.05) | 1.74 |
| Model 3 | 1.00  (reference) | 0.76  (0.53, 1.09) | 0.80  (0.56, 1.15) | 0.12 | 0.82  (0.63, 1.06) | 1.74 |
| Foods that are both ultra-processed and with front-of-package symbol, excluding food items with uncertain processing level or symbol eligibility |  |  |  |  |  |  |
| Mean ± SD | 12.7 ± 8.2 | 5.0 ± 0.9 | 2.0 ± 0.9 |  |  |  |
| Cases/person-years | 77/6,639 | 54/6,558 | 48/6,561 |  |  |  |
| Model 1 | 1.00  (reference) | 0.71  (0.50, 1.01) | 0.69  (0.48, 1.00) | 0.004 | 0.78  (0.66, 0.92) | 1.88 |
| Model 2 | 1.00  (reference) | 0.72  (0.50, 1.02) | 0.70  (0.48, 1.02) | 0.005 | 0.78  (0.66, 0.93) | 1.88 |
| Model 3 | 1.00  (reference) | 0.72  (0.51, 1.02) | 0.70  (0.48, 1.02) | 0.007 | 0.79  (0.66, 0.94) | 1.85 |

^1^ Model 1 was adjusted for age (years), sex (female, male), smoking status (never, past, current), household income (<$50,000; $50,000–$100,000; >$100,000), alcohol intake (g/day), physical activity level (low, moderate, high), hypertension status (none, unmedicated, medicated), and high blood cholesterol status (none, unmedicated, medicated). Model 2 was additionally adjusted for energy intake (kcal/day). Model 3 was additionally adjusted for energy intake (kcal/day) and body mass index (BMI; kg/m²).

^2^ *P*-values for trend were calculated by modeling the consumption of foods of concern as a continuous variable.

^3^ E-values were calculated using HRs for each 10% lower difference in intake.

Abbreviations: CI, confidence interval; HR, hazard ratio; SD, standard deviation.

# **Supplementary Table S8: Hazard ratios (HRs) and 95% confidence intervals (CIs) for cardiovascular disease risk according to consumption of foods of concerns (in percentage of grams per day) among individuals with hypertension (n=1,258).^1^**

|  | Tertile 3 | Tertile 2 | Tertile 1 | *P-*value for trend^2^ | HR (95% CI) for 10% lower difference in consumption | *E*-value^3^ |
| --- | --- | --- | --- | --- | --- | --- |
| Ultra-processed foods |  |  |  |  |  |  |
| Mean consumption ± SD | 26.7 ± 12.2 | 12.3 ± 2.0 | 5.4 ± 2.2 |  |  |  |
| Cases/person-years, n | 57/3,873 | 36/3,886 | 36/3,840 |  |  |  |
| Model 1 | 1.00 (reference) | 0.65  (0.42, 1.01) | 0.73  (0.47, 1.13) | 0.04 | 0.87  (0.76, 0.99) | 1.56 |
| Model 2 | 1.00 (reference) | 0.66  (0.43, 1.03) | 0.75  (0.47, 1.19) | 0.06 | 0.87  (0.76, 1.00) | 1.56 |
| Model 3 | 1.00 (reference) | 0.65  (0.42, 1.01) | 0.74  (0.47, 1.17) | 0.03 | 0.87  (0.75, 0.99) | 1.56 |
| Foods with front-of-package symbol |  |  |  |  |  |  |
| Mean consumption ± SD | 24.8 ± 7.1 | 13.6 ± 2.0 | 5.9 ± 2.6 |  |  |  |
| Cases/person-years, n | 57/3,875 | 39/3,869 | 33/3,855 |  |  |  |
| Model 1 | 1.00 (reference) | 0.71  (0.47, 1.06) | 0.66  (0.43, 1.03) | 0.003 | 0.75  (0.62, 0.91) | 2.00 |
| Model 2 | 1.00 (reference) | 0.71  (0.48, 1.08) | 0.68  (0.43, 1.07) | 0.006 | 0.75  (0.61, 0.92) | 2.00 |
| Model 3 | 1.00 (reference) | 0.71  (0.47, 1.08) | 0.68  (0.43, 1.07) | 0.007 | 0.76  (0.61, 0.93) | 1.96 |
| Foods that are ultra-processed and/or have a front-of-package symbol |  |  |  |  |  |  |
| Mean consumption ± SD | 33.8 ± 11.6 | 18.0 ± 2.6 | 8.2 ± 3.4 |  |  |  |
| Cases/person-years | 60/3,865 | 37/3,878 | 32/3,856 |  |  |  |
| Model 1 | 1.00 (reference) | 0.59  (0.39, 0.89) | 0.60  (0.37, 0.94) | 0.01 | 0.85  (0.75, 0.97) | 1.21 |
| Model 2 | 1.00 (reference) | 0.60  (0.39, 0.91) | 0.60  (0.37, 0.98) | 0.02 | 0.86  (0.75, 0.98) | 1.60 |
| Model 3 | 1.00 (reference) | 0.58  (0.38, 0.89) | 0.60  (0.37, 0.96) | 0.02 | 0.85  (0.74, 0.97) | 1.63 |
| Foods that are both ultra-processed and with front-of-package symbol |  |  |  |  |  |  |
| Mean consumption ± SD | 16.8 ± 6.7 | 8.5 ± 1.4 | 3.6 ± 1.5 |  |  |  |
| Cases/person-years | 55/3,879 | 35/3,912 | 39/3,809 |  |  |  |
| Model 1 | 1.00 (reference) | 0.66  (0.43, 1.01) | 0.84  (0.55, 1.28) | 0.006 | 0.73  (0.58, 0.91) | 2.08 |
| Model 2 | 1.00 (reference) | 0.67  (0.44, 1.03) | 0.86  (0.56, 1.34) | 0.01 | 0.73  (0.57, 0.93) | 2.08 |
| Model 3 | 1.00 (reference) | 0.67  (0.44, 1.03) | 0.86  (0.56, 1.34) | 0.01 | 0.73  (0.60, 0.93) | 2.08 |

^1^ Model 1 was adjusted for age (years), sex (female, male), smoking status (never, past, current), household income (<$50,000; $50,000–$100,000; >$100,000), alcohol intake (g/day), physical activity level (low, moderate, high), hypertension status (none, unmedicated, medicated), and high blood cholesterol status (none, unmedicated, medicated). Model 2 was additionally adjusted for energy intake (kcal/day). Model 3 was additionally adjusted for energy intake (kcal/day) and body mass index (BMI; kg/m²).

^2^ *P*-values for trend were calculated by modeling the consumption of foods of concern as a continuous variable.

^3^ E-values were calculated using HRs for each 10% lower difference in intake.

Abbreviations: CI, confidence interval; HR, hazard ratio; SD, standard deviation.

# **Supplementary Table S9: Hazard ratios (HRs) and 95% confidence intervals (CIs) for cardiovascular disease risk according to consumption of foods of concerns (in percentage of grams per day) among individuals with high blood cholesterol (n=1,334).^1^**

|  | Tertile 3 | Tertile 2 | Tertile 1 | *P* value for trend^2^ | HR (95% CI) for 10% lower difference in consumption | *E*-value^3^ |
| --- | --- | --- | --- | --- | --- | --- |
| Ultra-processed foods |  |  |  |  |  |  |
| Mean consumption ± SD | 28.7 ± 13.1 | 12.9 ± 2.1 | 5.8 ± 2.4 |  |  |  |
| Cases/person-years, n | 51/4,129 | 36/4,146 | 25/4,127 |  |  |  |
| Model 1 | 1.00 (reference) | 0.73  (0.47, 1.13) | 0.55  (0.34, 0.89) | 0.02 | 0.86  (0.76, 0.98) | 1.60 |
| Model 2 | 1.00 (reference) | 0.71  (0.46, 1.11) | 0.52  (0.32, 0.87) | 0.02 | 0.85  (0.75, 0.97) | 1.63 |
| Model 3 | 1.00 (reference) | 0.71  (0.46, 1.09) | 0.52  (0.31, 0.86) | 0.01 | 0.84  (0.74, 0.96) | 1.67 |
| Foods with front-of-package symbol |  |  |  |  |  |  |
| Mean consumption ± SD | 26.5 ± 9.4 | 14.0 ± 2.1 | 6.4 ± 2.7 |  |  |  |
| Cases/person-years, n | 50/4,163 | 36/4,104 | 26/4,135 |  |  |  |
| Model 1 | 1.00 (reference) | 0.72  (0.46, 1.11) | 0.58  (0.36, 0.94) | 0.004 | 0.78  (0.66, 0.92) | 1.88 |
| Model 2 | 1.00 (reference) | 0.70  (0.45, 1.09) | 0.56  (0.34, 0.92) | 0.003 | 0.78  (0.66, 0.92) | 1.88 |
| Model 3 | 1.00 (reference) | 0.70  (0.45, 1.08) | 0.56  (0.34, 0.93) | 0.004 | 0.78  (0.66, 0.92) | 1.88 |
| Foods that are ultra-processed and/or have a front-of-package symbol |  |  |  |  |  |  |
| Mean consumption ± SD | 35.7 ± 12.8 | 18.3 ± 2.6 | 8.4 ± 3.5 |  |  |  |
| Cases/person-years | 49/4,155 | 37/4,107 | 26/4,140 |  |  |  |
| Model 1 | 1.00 (reference) | 0.74  (0.48, 1.15) | 0.60  (0.37, 0.97) | 0.01 | 0.86  (0.77, 0.97) | 1.60 |
| Model 2 | 1.00 (reference) | 0.72  (0.46, 1.13) | 0.57  (0.35, 0.95) | 0.01 | 0.85  (0.76, 0.96) | 1.63 |
| Model 3 | 1.00 (reference) | 0.70  (0.44, 1.10) | 0.57  (0.34, 0.94) | 0.003 | 0.84  (0.74, 0.95) | 1.67 |
| Foods that are both ultra-processed and with front-of-package symbol |  |  |  |  |  |  |
| Mean consumption ± SD | 18.8 ± 9.0 | 9.1 ± 1.3 | 4.0 ± 1.7 |  |  |  |
| Cases/person-years | 47/4,180 | 38/4,092 | 27/4,130 |  |  |  |
| Model 1 | 1.00 (reference) | 0.89  (0.57, 1.37) | 0.66  (0.41, 1.06) | 0.003 | 0.75  (0.62, 0.91) | 2.00 |
| Model 2 | 1.00 (reference) | 0.87  (0.56, 1.35) | 0.64  (0.39, 1.05) | 0.003 | 0.75  (0.62, 0.90) | 2.00 |
| Model 3 | 1.00 (reference) | 0.87  (0.56, 1.35) | 0.65  (0.40, 1.06) | 0.003 | 0.75  (0.62, 0.91) | 2.00 |

^1^ Model 1 was adjusted for age (years), sex (female, male), smoking status (never, past, current), household income (<$50,000; $50,000–$100,000; >$100,000), alcohol intake (g/day), physical activity level (low, moderate, high), hypertension status (none, unmedicated, medicated), and high blood cholesterol status (none, unmedicated, medicated). Model 2 was additionally adjusted for energy intake (kcal/day). Model 3 was additionally adjusted for energy intake (kcal/day) and body mass index (BMI; kg/m²).

^2^ *P*-values for trend were calculated by modeling the consumption of foods of concern as a continuous variable.

^3^ E-values were calculated using HRs for each 10% lower difference in intake.

Abbreviations: CI, confidence interval; HR, hazard ratio; SD, standard deviation.

# **Supplementary Table S10: Characteristics of the 1,258 participants with hypertension according to BP-lowering medication use.^1^**

| **Characteristics** | **Participants not using BP-lowering medication (n=283)** | **Participants using BP-lowering medication (n=975)** |
| --- | --- | --- |
| Age, years | 55.0 ± 7.4 | 58.1 ± 7.2 |
| Sex, n (%) |  |  |
| Female | 146 (51.6) | 513 (52.6) |
| Male | 137 (48.4) | 462 (47.4) |
| Annual household income, n (%) |  |  |
| <$50,000 | 85 (30.0) | 325 (33.3) |
| $50,000<$100,000 | 117 (41.3) | 420 (43.1) |
| ≥$100,000 | 81 (28.6) | 230 (23.6) |
| Smoking status, n (%) |  |  |
| Never | 116 (41.0) | 407 (41.7) |
| Past | 128 (45.2) | 441 (45.2) |
| Current | 39 (13.8) | 127 (13.0) |
| Body mass index, kg/m² | 28.3 ± 5.7 | 29.5 ± 6.0 |
| Physical activity level, n (%) |  |  |
| Low | 39 (13.8) | 162 (16.6) |
| Moderate | 124 (43.8) | 365 (37.4) |
| High | 120 (42.4) | 448 (46.0) |
| High blood cholesterol, n (%) | 80 (28.3) | 389 (39.9) |
| Cholesterol-lowering medication use, n (%) | 29 (10.3) | 279 (28.6) |
| Statin (C10AA) | 28 (9.9) | 271 (27.8) |
| Ezetimibe (C10AX09) | 1 (0.4) | 18 (1.9) |
| Combination therapy for hypercholesterolemia | 1 (0.4) | 10 (1.0) |
| Antihypertensive medication | 0 | 975 (100) |
| Antihypertensives (C02) | 0 | 21 (2.2) |
| Diuretics (C03) | 0 | 190 (19.5) |
| Beta blocking agents (C07) | 0 | 213 (21.9) |
| Calcium channel blockers (C08) | 0 | 200 (20.5) |
| Agents acting on the renin-angiotensin system (C09) | 0 | 661 (67.8) |
| Combination therapy for hypertension | 0 | 271 (27.8) |
| Blood pressure, mm Hg |  |  |
| Systolic | 137 ± 17 | 130 ± 15 |
| Diastolic | 82 ± 11 | 77 ± 10 |
| Plasma lipids, mmol/L |  |  |
| Total-cholesterol | 5.40 ± 0.97 | 5.11 ± 1.01 |
| Triglycerides | 1.93 ± 1.02 | 2.04 ± 1.39 |
| LDL-cholesterol | 3.26 ± 0.82 | 3.01 ± 0.85 |
| HDL-cholesterol | 1.25 ± 0.38 | 1.20 ± 0.38 |
| Framingham risk score, percentage | 11.5 ± 3.8 | 11.6 ± 3.3 |
| Dietary intakes |  |  |
| Alternate healthy eating index, points | 49.9 ± 10.5 | 49.1 ± 10.7 |
| Energy intake, kcal per day | 1,900 ± 701 | 1,886 ± 690 |
| Saturated fats, percentage of calories per day | 10.5 ± 2.7 | 10.6 ± 2.8 |
| Unsaturated fats, percentage of calories per day | 19.6 ± 4.3 | 19.4 ± 4.3 |
| Sodium (mg/day) | 2,693 ± 1,065 | 2,804 ± 1,174 |
| Sugar, percentage of calories per day | 22.2 ± 7.4 | 22.3 ± 7.7 |
| Fiber, grams per day | 20.5 ± 10.5 | 19.6 ± 8.7 |
| Alcohol consumption, grams per day | 12.5 ± 17.7 | 11.6 ± 19.6 |
| Ultra-processed food intake (percentage of grams per day) | 14.4 ± 11.5 | 15.1 ± 11.5 |
| Foods with front-of-package nutrition symbol (percentage of grams per day) | 14.5 ± 9.2 | 15.0 ± 9.0 |

^1^ Continuous variables are presented as mean ± standard deviation. Categorical variables are presented as count (percentage)

Abbreviation: BP, blood pressure.

# **Supplementary Table S11: Characteristics of the 1,334 participants with high blood cholesterol according to cholesterol-lowering medication use.^1^**

| **Characteristics** | **Participants not using cholesterol-lowering medication (n=621)** | **Participants using cholesterol-lowering medication (n=713)** |
| --- | --- | --- |
| Age, years | 54.6 ± 7.4 | 58.7 ± 6.8 |
| Sex, n (%) |  |  |
| Female | 308 (49.6) | 315 (44.2) |
| Male | 313 (50.4) | 398 (55.8) |
| Annual household income, n (%) |  |  |
| <$50,000 | 190 (30.6) | 215 (30.2) |
| $50,000<$100,000 | 259 (41.7) | 321 (45.0) |
| ≥$100,000 | 172 (27.7) | 177 (24.8) |
| Smoking status, n (%) |  |  |
| Never | 243 (39.1) | 254 (35.6) |
| Past | 278 (44.8) | 347 (48.7) |
| Current | 100 (16.1) | 112 (15.7) |
| Body mass index, kg/m² | 27.7 ± 5.0 | 28.7 ± 4.6 |
| Physical activity level, n (%) |  |  |
| Low | 102 (16.4) | 135 (18.9) |
| Moderate | 240 (38.7) | 249 (34.9) |
| High | 279 (44.9) | 329 (46.1) |
| Cholesterol-lowering medication use, n (%) | 0 | 713 (100) |
| Statin (C10AA) | 0 | 696 (97.6) |
| Ezetimibe (C10AX09) | 0 | 37 (5.2) |
| Combination therapy for hypercholesterolemia | 0 | 20 (2.8) |
| Hypertension, n (%) | 161 (25.9) | 308 (43.2) |
| Antihypertensive medication use, n (%) | 110 (17.7) | 279 (39.1) |
| Antihypertensives (C02) | 4 (0.6) | 3 (0.4) |
| Diuretics (C03) | 20 (3.2) | 43 (6.0) |
| Beta blocking agents (C07) | 24 (3.9) | 74 (10.4) |
| Calcium channel blockers (C08) | 22 (3.5) | 66 (9.3) |
| Agents acting on the renin-angiotensin system (C09) | 69 (11.1) | 202 (28.3) |
| Combination therapy for hypertension | 27 (4.4) | 95 (13.3) |
| Blood pressure, mm Hg |  |  |
| Systolic | 125 ± 15 | 128 ± 15 |
| Diastolic | 75 ± 10 | 75 ± 10 |
| Plasma lipids, mmol/L |  |  |
| Total-cholesterol | 5.88 ± 0.97 | 4.60 ± 0.88 |
| Triglycerides | 2.22 ± 1.79 | 2.13 ± 1.48 |
| LDL-cholesterol | 3.70 ± 0.80 | 2.52 ± 0.71 |
| HDL-cholesterol | 1.19 ± 0.41 | 1.16 ± 0.34 |
| Framingham risk score, percentage | 11.0 ± 3.5 | 11.3 ± 3.2 |
| Dietary intakes |  |  |
| Alternate healthy eating index, points | 51.2 ± 10.9 | 49.2 ± 11.2 |
| Energy intake, kcal per day | 1,944 ± 715 | 1,895 ± 687 |
| Saturated fats, percentage of calories per day | 10.2 ± 2.6 | 10.5 ± 2.6 |
| Unsaturated fats, percentage of calories per day | 19.5 ± 4.4 | 19.4 ± 4.2 |
| Sodium (mg/day) | 2,823 ± 1,158 | 2,811 ± 1,130 |
| Sugar, percentage of calories per day | 23.0 ± 7.9 | 22.2 ± 7.7 |
| Fiber, grams per day | 21.1 ± 9.6 | 19.6 ± 9.0 |
| Alcohol consumption, grams per day | 10.4 ± 15.3 | 12.9 ± 21.7 |
| Ultra-processed food intake (percentage of grams per day) | 15.5 ± 12.1 | 16.3 ± 12.7 |
| Foods with front-of-package nutrition symbol (percentage of grams per day) | 15.6 ± 10.7 | 15.9 ± 9.7 |

^1^ Continuous variables are presented as mean ± standard deviation. Categorical variables are presented as count (percentage)

**Supplementary Table S12: Hazard ratios (HRs) and 95% confidence intervals (Cis) for cardiovascular disease risk according to medication status.^1^**

| **Model** | **Unmedicated** | **Medicated** | ***P*-value** |
| --- | --- | --- | --- |
| Hypertension |  |  |  |
| Participants, n | N=283 | N=975 |  |
| Cases/person-years, n | 26/2,614 | 103/8,986 |  |
| Hazard ratio (95% confidence interval) | 1.00  (reference) | 1.12  (0.71, 1.75) | 0.62 |
| High blood cholesterol |  |  |  |
| Participants, n | N=621 | N=713 |  |
| Cases/person-years, n | 57/5,738 | 55/6,664 |  |
| Hazard ratio (95% confidence interval) | 1.00  (reference) | 0.59  (0.39, 0.89) | 0.01 |

^1^ Analysis on hypertension medication included n=1,258 participants with hypertension. Analysis on high blood cholesterol medication included n=1,334 participants with high blood cholesterol. Model was adjusted for age (years, sex (female, male), household income (<$50,000; $50,000–$100,000; >$100,000), alcohol intake (g/day), physical activity level (low, moderate, high), smoking status (never, past, current), energy intake (kcal/day), ultra-processed food intake (percentage of grams per day), body mass index (kg/m^2^), and concomitant high blood cholesterol (none, unmedicated, medicated; for the analysis on hypertension) or concomitant hypertension (none, unmedicated, medicated; for the analysis on high blood cholesterol).

# **Supplementary Table S13: Differences in blood pressure and LDL-cholesterol according to medication status at baseline.**

| **Model** | **Unmedicated** | **Medicated** | ***P*-value** |
| --- | --- | --- | --- |
| Participants with hypertension^1^ |  |  |  |
| Systolic blood pressure, mm Hg | 138  (136, 140) | 130  (129, 131) | <0.0001 |
| Diastolic blood pressure, mm Hg | 82  (80, 83) | 77  (76, 78) | <0.0001 |
| Participants with high blood cholesterol^2^ |  |  |  |
| LDL-cholesterol, mmol/L | 3.67  (3.59, 3.75) | 2.54  (2.46, 2.62) | <0.0001 |

Analyses were was adjusted for age (years), sex (female, male), smoking status (never, past, current), household income (<$50,000; $50,000–$100,000; >$100,000), alcohol intake (g/day), physical activity level (low, moderate, high), ultra-processed food intake (percentage of grams per day), energy intake (kcal/day), body mass index (BMI; kg/m²) and concomitant high blood cholesterol (none, unmedicated, medicated; for analyses on blood pressure) or concomitant hypertension (none, unmedicated, medicated; for analysis on LDL-C). Data are presented as mean (95% confidence interval).

^1^ Analyses on systolic and diastolic blood pressures included n=1,258 participants with hypertension, of that 975 were using blood pressure-lowering medication.

^2^ Analysis on LDL-cholesterol included n=1,334 participants with high blood cholesterol, of that 713 were using cholesterol-lowering medication.

**
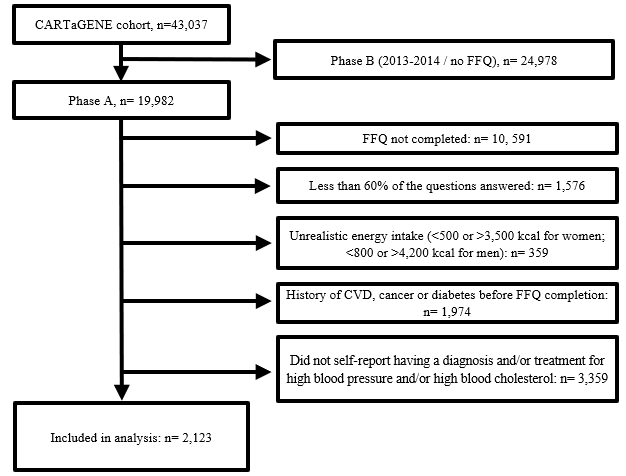
**

# **Supplementary Figure S1: Flow-chart of participants’ selection.**

Abbreviations: CVD, cardiovascular disease; FFQ, food frequency questionnaire.

**
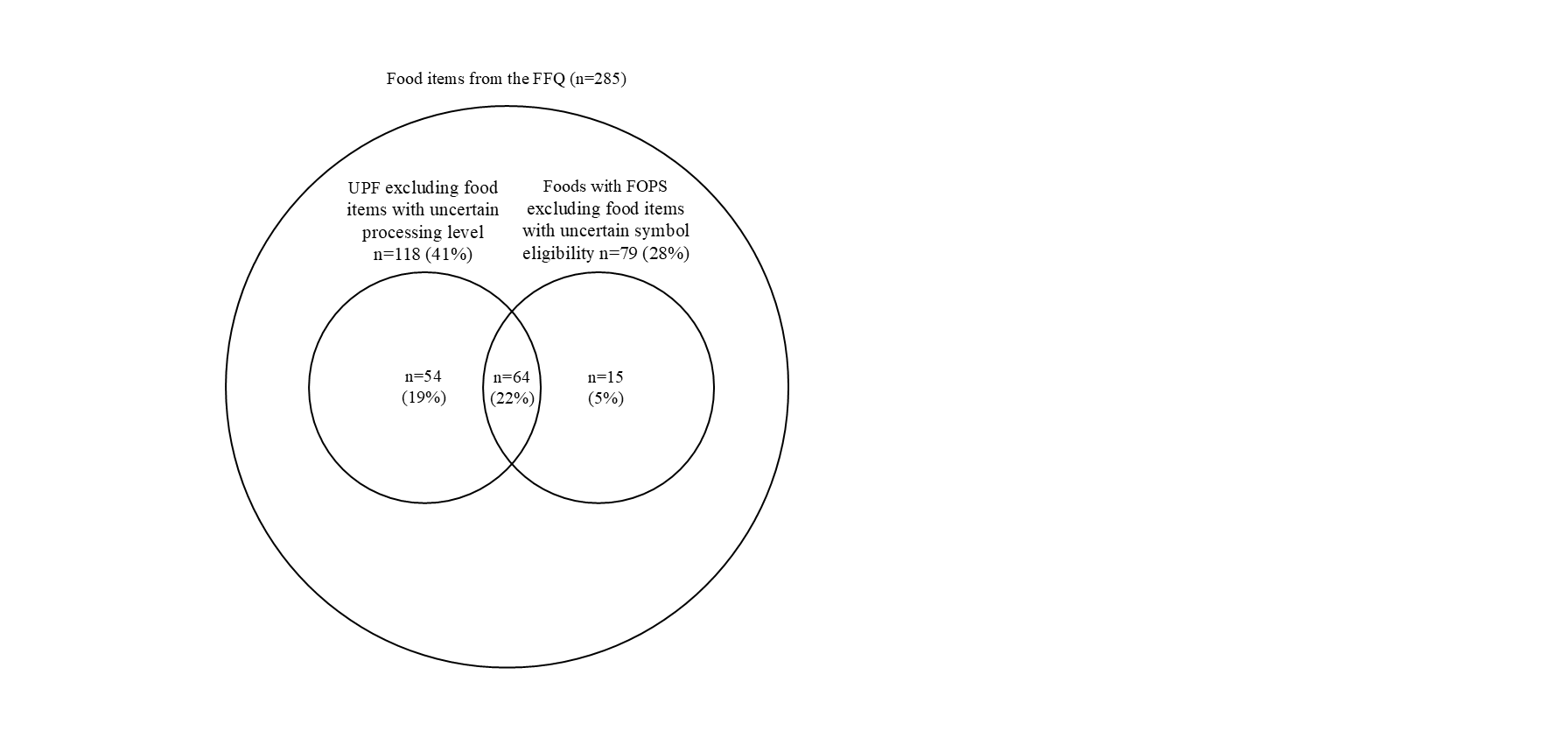
**

# **Supplementary Figure S2: Distribution of the 285 food items from the Canadian dietary history questionnaire II across the foods with front-of-package nutrition symbol and ultra-processed foods classifications, excluding food items with uncertain category.**

Abbreviations: FFQ, food frequency questionnaire; FOPS**,** front-of-package nutrition symbol; UPF, ultra-processed foods.


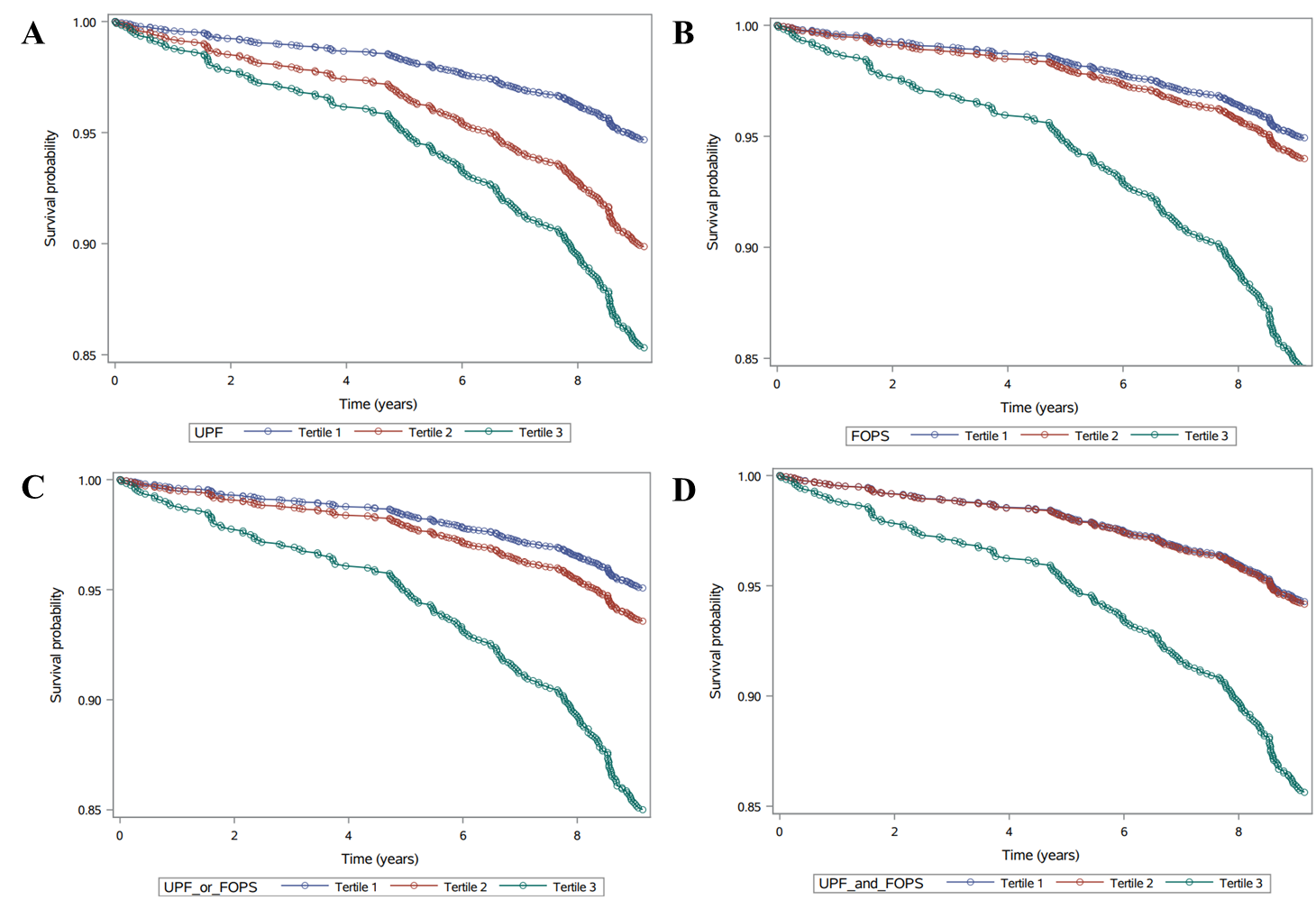


# **Supplementary Figure S3: Survival curves for the association between consumption of AA) ultra-processed foods (UPF); (B) foods with a front-of-package symbol (FOPS); (C) foods that are ultra-processed or with a FOPS; (D) foods that are both ultra-processed and with a FOPS.**

Abbreviations: FOPS**,** front-of-package nutrition symbol; UPF, ultra-processed foods.
